# Lung microbiota composition, respiratory mechanics, and outcomes in COVID-19-related ARDS

**DOI:** 10.1128/spectrum.03574-23

**Published:** 2024-03-11

**Authors:** Gennaro De Pascale, Brunella Posteraro, Flavio De Maio, Pia Clara Pafundi, Eloisa Sofia Tanzarella, Salvatore Lucio Cutuli, Gianmarco Lombardi, Domenico Luca Grieco, Emanuele Franchini, Giulia Santarelli, Amato Infante, Maurizio Sanguinetti, Massimo Antonelli

**Affiliations:** 1 Dipartimento di Scienze Biotecnologiche di Base, Cliniche Intensivologiche e Perioperatorie, Università Cattolica del Sacro Cuore, Rome, Italy; 2 Dipartimento di Scienze dell'Emergenza, Anestesiologiche e della Rianimazione, Fondazione Policlinico Universitario A. Gemelli IRCCS, Rome, Italy; 3 Dipartimento di Scienze Mediche e Chirurgiche, Fondazione Policlinico Universitario A. Gemelli IRCCS, Rome, Italy; 4 Dipartimento di Scienze di Laboratorio e Infettivologiche, Fondazione Policlinico Universitario A. Gemelli IRCCS, Rome, Italy; 5 Epidemiology and Biostatistics Research Core Facility, Gemelli Science & Technology Park, Fondazione Policlinico Universitario A. Gemelli IRCCS, Rome, Italy; 6 Dipartimento di Scienze Radiologiche ed Ematologiche, Fondazione Policlinico Universitario A. Gemelli IRCCS, Rome, Italy; University of Arkansas Fayetteville, Fayetteville, Arkansas, USA

**Keywords:** ARDS, COVID-19, mortality, microbiota, respiratory mechanics

## Abstract

Few data are available on the lung microbiota composition of patients with coronavirus disease 2019-related acute respiratory distress syndrome (C-ARDS) receiving invasive mechanical ventilation (IMV). Moreover, it has never been investigated whether there is a potential correlation between lung microbiota communities and respiratory mechanics. We performed a prospective observational study in two intensive care units of a university hospital in Italy. Lung microbiota was investigated by bacterial 16S rRNA gene sequencing, performed on bronchoalveolar lavage fluid samples withdrawn after intubation. The lung bacterial communities were analyzed after stratification by respiratory system compliance/predicted body weight (Crs) and ventilatory ratio (VR). Weaning from IMV and hospital survival were assessed as secondary outcomes. In 70 C-ARDS patients requiring IMV from 1 April through 31 December 2020, the lung microbiota composition (phylum taxonomic level, permutational multivariate analysis of variance test) significantly differed between who had low Crs vs those with high Crs (*P* = 0.010), as well as in patients with low VR vs high VR (*P* = 0.012). As difference-driving taxa, Proteobacteria (*P* = 0.017) were more dominant and Firmicutes (*P* = 0.040) were less dominant in low- vs high-Crs patients. Similarly, Proteobacteria were more dominant in low- vs high-VR patients (*P* = 0.013). After multivariable regression analysis, we further observed lung microbiota diversity as a negative predictor of weaning from IMV and hospital survival (hazard ratio = 3.31; 95% confidence interval, 1.52–7.20, *P* = 0.048). C-ARDS patients with low Crs/low VR had a Proteobacteria-dominated lung microbiota. Whether patients with a more diverse lung bacterial community may have more chances to be weaned from IMV and discharged alive from the hospital warrants further large-scale investigations.

**IMPORTANCE:**

Lung microbiota characteristics were demonstrated to predict ventilator-free days and weaning from mechanical ventilation in patients with acute respiratory distress syndrome (ARDS). In this study, we observed that in severe coronavirus disease 2019 patients with ARDS who require invasive mechanical ventilation, lung microbiota characteristics were associated with respiratory mechanics. Specifically, the lung microbiota of patients with low respiratory system compliance and low ventilatory ratio was characterized by Proteobacteria dominance. Moreover, after multivariable regression analysis, we also found an association between patients’ microbiota diversity and a higher possibility of being weaned from mechanical ventilation and discharged alive from the hospital. For these reasons, lung microbiota characterization may help to stratify patient characteristics and orient the delivery of target interventions. (This study has been registered at ClinicalTrials.gov on 17 February 2020 under identifier NCT04271345.)

**Clinical Trial:**

Registered at ClinicalTrials.gov, 17 February 2020 (NCT0427135).

## INTRODUCTION

Acute respiratory distress syndrome (ARDS) is a common though heterogeneous disease leading to high mortality and costs (1), whose burden has been recently exacerbated by the coronavirus disease 2019 (COVID-19) pandemic (2). The COVID-19-related ARDS (C-ARDS) (3) is characterized by physiological abnormalities of typical ARDS (2), which include inflammatory alveolar flooding and interstitial infiltrations leading to reduced respiratory system compliance (Crs) (4), and capillary thrombosis fostering pulmonary dead space increase, which directly correlates with the ventilatory ratio (VR) (5–8). In critically ill patients with non-COVID-19 (9, 10) and COVID-19 (11) ARDS requiring invasive mechanical ventilation (IMV), both of these conditions were reported to predict mortality. Furthermore, recent evidence showed that inflammatory exudate that characterized ARDS lungs acts as a nutrient-enriched microbiological ground that, in conjunction with altered local O_2_ and CO_2_ tension, influences the growth of certain microbiota communities, thus triggering additional immune response and injury (6). In critical care practice, lung microbiota composition was demonstrated to correlate with ventilator-free days (12) in a general population of critically ill patients, as well as it predicts the successful extubation and mortality in those with COVID-19 ARDS (13).

In light of the above considerations, we formulated the hypothesis that different lung microbiota features may correlate with specific respiratory mechanics phenotypes. Accordingly, we prospectively investigated the relationship between the heterogeneity of lung microbiota (diversity and composition) and respiratory system mechanics assessed via Crs and VR in critically ill patients with COVID-19 ARDS. Moreover, as a secondary outcome and proof of concept, we explored potential relationships between lung microbiota composition and relevant clinical outcomes like successful weaning from IMV and hospital survival in those intensive care unit (ICU) patients.

## RESULTS

### C-ARDS patient cohort description

We studied 70 mechanically ventilated C-ARDS patients consecutively admitted to the ICU (Table 1; eTable 1). Lung microbiota analysis (eFigure 2) was performed on the first bronchoalveolar lavage (BAL) obtained after intubation. Mean (SD) age was 67.2 ± 9 years, males were prevalent (*n* = 49; 70%), and the most frequent comorbidity was hypertension (35/70, 50%), while 6 (8.6%) patients had chronic obstructive pulmonary disease. Serum D-dimer and lactate dehydrogenase (LDH) were elevated [median interquartile (IQR) values, 3,540 (1,074–4,314) ng/mL and 319.5 (259.0–399.8) IU/L, respectively], whereas lymphocyte count and serum procalcitonin (PCT) were low [median (IQR) values of 1.1 (0.7–1.6) × 10^9^/L and 0.34 (0.16–0.84) ng/mL, respectively]. Mean (SD) Crs was 0.53 ± 0.17 (mL/cmH_2_O)/kg, whereas median (IQR) VR was 2.2 (1.8–2.5). Thirty-four (48.6%) patients died in the ICU, 39 (55.7%) at hospital discharge, and none of them were weaned from IMV. Only three patients underwent veno-venous extracorporeal-membrane oxygenation treatment, and they all survived. Of note, all patients discharged alive from the hospital were weaned from IMV. The median (IQR) hospital length of stay (LoS) was 31 (IQR 18–54) days.

**TABLE 1:** Characteristics of COVID-19 patients who received mechanical ventilation for ARDS in the ICU, both overall and stratified compliance and VR subgroups (*n* = 70)^*a*^

		Compliance of respiratory system/PBW^*c*^			VR^*c*^		
Variables^*b*^	Total (*n* = 70)	Low (*n* = 37)	High (*n* = 33)	*P*	Low (*n* = 36)	High (*n* = 34)	*P*
Age (years)	67.2 (9.0)	67.3 (9.3)	67.0 (8.7)	0.892	65.2 (9.7)	69.3 (7.7)	*0.058*
Sex				**0.030**			1.000
*Male*	49 (70)	20 (54.1)	29 (87.9)		25 (69.4)	24 (70.6)	
*Female*	21 (30)	17 (45.9)	4 (12.1)		11 (30.6)	10 (29.4)	
Comorbidities							
*Hypertension*	35 (50)	18 (48.6)	17 (51.5)	1.000	18 (50)	17 (50)	1.000
*Coronary artery disease*	14 (20)	5 (13.5)	9 (27.3)	0.231	9 (14)	5 (14.7)	0.374
*Immunosuppression*	14 (20)	8 (21.6)	6 (18.2)	0.772	7 (19.4)	7 (20.6)	1.000
*Diabetes mellitus*	12 (17.1)	6 (16.2)	6 (18.2)	1.000	7 (19.4)	5 (14.7)	0.754
*Neoplasm*	8 (11.4)	5 (13.5)	3 (9.1)	0.714	4 (11.1)	4 (11.8)	1.000
*Chronic heart disease*	7 (10)	3 (8.1)	4 (12.1)	0.699	5 (13.9)	2 (5.9)	0.430
*COPD*	6 (8.6)	3 (8.1)	3 (9.1)	1.000	3 (8.3)	3 (8.8)	1.000
*Chronic vasculopathy*	4 (5.7)	3 (8.1)	1 (3.0)	0.616	2 (5.6)	2 (5.9)	1.000
Charlson comorbidity index	2 (1–4)	2 (1–3)	2 (1–4)	0.872	2 (1–3)	3 (1–4)	**<0.001**
SAPS II	40.2 (16.7)	37.6 (15.7)	43.1 (17.5)	0.173	35.7 (16.5)	45.1 (15.8)	**0.017**
Previous hospitalization	22 (31.4)	13 (35.1)	9 (27.3)	0.607	11 (30.6)	11 (32.4)	1.000
Days from the onset of symptoms	13.5 (8.1)	13.5 (8.1)	13.4 (8.1)	0.953	12.7 (8.8)	14.3 (7.3)	0.384
Days from the start of MV	3 (2–8)	3 (2–9)	3 (2–8)	**<0.001**	2.5 (2.0–9.0)	4.0 (2.0–7.7)	**<0.001**
C-ARDS clinical/laboratory variables							
Steroid therapy receipt	32 (45.7)	17 (45.9)	15 (45.5)	1.000	14 (38.9)	18 (52.9)	0.337
Interleukin-6 inhibitor receipt	10 (14.3)	2 (5.4)	8 (24.2)	**0.038**	1 (2.8)	9 (26.5)	**0.006**
Antibiotics receipt^*d*^	50 (70)	25 (67.6)	25 (75.8)	0.6	25 (69.4)	25 (73.5)	**0.79**
White blood cell count (10^9^/L)	14.2 (6.0)	14.0 (5.8)	14.5 (6.4)	0.720	13.5 (6.0)	15.0 (6.0)	0.304
Lymphocyte count (10^9^/L)	1.1 (0.7–1.6)	1.2 (0.6–1.7)	1.0 (0.7–1.4)	**0.036**	1.2 (0.6–1.6)	1.0 (0.7–1.5)	**0.033**
Neutrophil count (10^9^/L)	12.0 (5.9)	11.5 (5.8)	12.5 (6.1)	0.497	11.5 (5.7)	12.5 (6.2)	0.474
D-dimer (ng/mL)	3,540 (1,074–4,314)	3,554 (1,206–3,981)	3,224 (909–4,979)	**<0.001**	1,700 (841–4,255)	3,726 (2,140–4,346)	**<0.001**
Fibrinogen (ng/mL)	650.8 (253.4)	697.4 (250.4)	598.5 (250.3)	0.104	685.7 (251.4)	613.8 (253.9)	0.238
Lactate dehydrogenase (IU/L)	319.5 (259.0–399.8)	315 (265–380)	326 (255–508)	**<0.001**	334.5 (267.8–413.0)	302.0 (246.5–375.8)	**<0.001**
Procalcitonin (ng/mL)	0.34 (0.16–0.84)	0.54 (0.18–1.13)	0.27 (0.12–0.75)	**<0.001**	0.38 (0.16–1.21)	0.28 (0.16–0.67)	**<0.001**
CRP (mg/L)	168.7 (203.9)	160.4 (103.5)	178.1 (184.5)	0.732	186.0 (270.1)	150.4 (94.3)	0.461
PaO_2_/FiO_2_ ratio (mmHg)	142.0 (122.2–183.8)	132 (124–168)	160 (122–198)	**<0.001**	138.5 (123.5–191.8)	146.0 (120.8–179.2)	0.697
PaCO_2_ (mmHg)	49.8 (11.0)	47.8 (10.2)	52.0 (11.7)	0.116	43.7 (7.7)	56.2 (10.4)	**<0.001**
Oxygen saturation index	11.8 (9.2–17.0)	9.8 (8.7–11.9)	16.1 (11.8–19.6)	**<0.001**	11.4 (9.2–15.0)	13.3 (9.5–17.2)	**<0.001**
Respiratory mechanics measures							
Tidal volume/PBW (mL/kg)	6.0 (5.4–6.8)	5.6 (5.2–6.4)	6.5 (6.0–7.2)	**<0.001**	5.5 (5.2–13.8)	6.4 (5.8–7.1)	**<0.001**
Respiratory rate (bpm)	27.7 (4.4)	27.9 (4.8)	27.5 (4.0)	0.700	26.2 (4.4)	29.3 (3.8)	**0.002**
PEEP (cmH_2_O)	10 (8–14)	10 (8–12)	13 (8–15)	**<0.001**	11.0 (8.0–13.2)	10.0 (8.0–14.7)	0.794
*P*_PLAT_ (cmH_2_O)	23.2 (3.6)	24.5 (3.2)	21.8 (3.4)	**0.001**	23.6 (3.9)	22.8 (3.2)	0.321
Driving pressure (cmH_2_O)	12.5 (3.7)	14.7 (3.5)	10.1 (2.1)	**<0.001**	12.8 (3.7)	12.2 (3.8)	0.486
Recruitment to inflation ratio	0.56 (0.38)	0.49 (0.30)	0.65 (0.44)	0.100	0.65 (0.45)	0.48 (0.26)	*0.051*
Corrected minute ventilation (L)	13.9 (5.2)	11.8 (3.3)	16.4 (5.8)	**<0.001**	10.8 (2.9)	17.3 (5.0)	**<0.001**
Crs (mL/cmH_2_O)	35.2 (13.1)	25.5 (7.0)	46.2 (9.1)	**<0.001**	32.2 (11.3)	38.4 (14.3)	**0.047**
ELrs/PBW ([cmH_2_O/mL]/kg)	1.9 (1.6–2.4)	2.7 (2.1–3.1)	1.6 (1.4–1.7)	**<0.001**	2.0 (1.8–2.6)	1.7 (1.4–2.2)	**<0.001**
Crs/PBW ([mL/cmH_2_O]/kg)	0.53 (0.17)	–	–		–	–	
VR	2.2 (1.8–2.5)	–	–		–	–	
Outcomes							
Steroids duration (days)	10 (7–10)	10 (7–10)	10 (7–10)	0.519	10.0 (5.5–10.0)	10.0 (9.2–10.0)	**<0.001**
NIV-HFO preBAL				0.625			1.000
*Yes*	43 (61.4)	19 (57.6)	24 (64.9)		22 (61.1)	21 (61.8)	
*No*	27 (38.6)	14 (42.4)	13 (35.1)		14 (38.9)	13 (38.2)	
NIV-HFO preBAL days^*e*^	5 (2–9)	8 (3–9)	4.0 (1.7–7.2)	**<0.001**	4.0 (1.0–7.5)	7 (3–9)	**<0.001**
28-day weaning from MV	29 (41.4)	11 (29.7)	18 (54.5)	* 0.052*	17 (47.2)	12 (35.3)	0.341
Overall weaning from MV	31 (44.3)	13 (35.1)	18 (54.5)	*0.057*	18 (50)	13 (38.2)	0.700
Days of ICU LoS	19.0 (13.0–36.7)	20 (12–44)	19 (14–36)	**<0.001**	20.5 (13.0–37.5)	19 (13.2–31.7)	0.911
Days of hospital LoS	31 (18–54)	25 (16–53)	38 (20–54)	**<0.001**	34.5 (19.7–54.0)	25.5 (16.0–52.7)	**<0.001**
28-day mortality	34 (48.6)	23 (62.2)	11 (33.3)	**0.019**	15 (41.7)	19 (55.9)	0.339
In-hospital mortality	39 (55.7)	24 (64.9)	15 (45.5)	*0.081*	18 (50)	21 (61.8)	0.300

^*a*^ Qualitative data are expressed as absolute and relative percentage frequency, and *P* values were computed by Fisher–Freeman–Halton’s exact test. Quantitative data are instead described as either mean and SD or median and IQR, as appropriate. Between-group comparisons were assessed by either Student’s *t* test or the Wilcoxon–Mann–Whitney *U* test (Wilcoxon–Pratt signed rank test in the presence of ties). *P* values on survival and weaning data were computed instead by log-rank test. All variables, except for outcomes, were assessed at the time patients underwent bronchoalveolar fluid sampling. Significant findings are indicated in bold (*P* < 0.05), and the suggestive ones are in italic (0.05 ≤ *P* < 0.10).

^*b*^ COPD, chronic obstructive pulmonary disease; PBW, predicted body weight; SAPS, simplified acute physiology score; MV, mechanical ventilation; CRP, C-reactive protein; PaO_2_/FiO_2_, partial pressure of arterial oxygen/fraction of inspired oxygen; PaCO_2_, arterial partial pressure of carbon dioxide; PEEP, positive end-expiratory pressure; *P*_PLAT_, plateau pressure; ELrs, elastance of the respiratory system.

^*c*^ Both Crs/PBW and VR values were assessed on the day of bronchoalveolar fluid sampling. Patients were stratified on the median value as low-Crs (Crs/PBW values, ≤0.53) or high-Crs (Crs/PBW values, >0.53) patients and as low-VR (VR values, ≤2.2) or high-VR (VR values, >2.2) patients.

^*d*^ Low Crs: macrolides (*n* = 7), cephalosporins (*n* = 4), Beta-lactam-inhibitor combination (BLIC *, n* = 6), tigecycline (*n* = 4), linezolid (*n* = 3), and quinolones (*n* = 1); high Crs: macrolides (*n* = 9), cephalosporins (*n* = 8), BLIC (*n* = 4), tigecycline (*n* = 1), linezolid (*n* = 2), and quinolones (*n* = 1); low VR: macrolides (*n* = 9), cephalosporins (*n* = 3), BLIC (*n* = 7), tigecycline (*n* = 2), linezolid (*n* = 4), and quinolones (*n* = 0); high VR: macrolides (*n* = 7), cephalosporins (*n* = 9), BLIC (*n* = 3), tigecycline (*n* = 3), linezolid (*n* = 1), and quinolones (*n* = 2).

^*e*^ Calculations were performed only on patients who experimented a non invasive ventilation-high flow oxygen (NIV-HFO) event.

### C-ARDS patient stratification by respiratory mechanics variables

Patients were stratified by mean Crs and median VR (Tables 1; eTable 2). Patients with low Crs showed higher D-dimer (*P* < 0.001), higher PCT (*P* < 0.001), lower LDH (*P* < 0.001), lower oxygen saturation index (*P* < 0.001), lower PaO_2_/FiO_2_ (*P* < 0.001), lower tidal volumes (*P* < 0.001), lower positive end-expiratory pressure (*P* < 0.001), higher driving pressure (*P* < 0.001), longer ICU LoS (*P* < 0.001), and had higher 28-day mortality (*P* = 0.019) as compared to patients with high Crs. Hospital mortality was not different. Patients with low VR showed lower D-dimer (*P* < 0.001), higher PCT (*P* < 0.001), higher LDH (*P* < 0.001), lower PaCO_2_ (*P* < 0.001), lower tidal volumes (*P* > 0.001), lower respiratory rate (*P* < 0.002), lower oxygen saturation index (*P* < 0.001), and longer ICU LoS (*P* < 0.001) as compared to patients with high VR, but 28-day and hospital mortality did not differ. We further assessed the cumulative incidence of the primary outcome by a Kaplan–Meier (KM) curve analysis, stratifying patients for Crs and VR (eFigre 3), and found no significant differences between these groups.

### Lung microbiota analysis in C-ARDS patient groups

**Fig 1:**
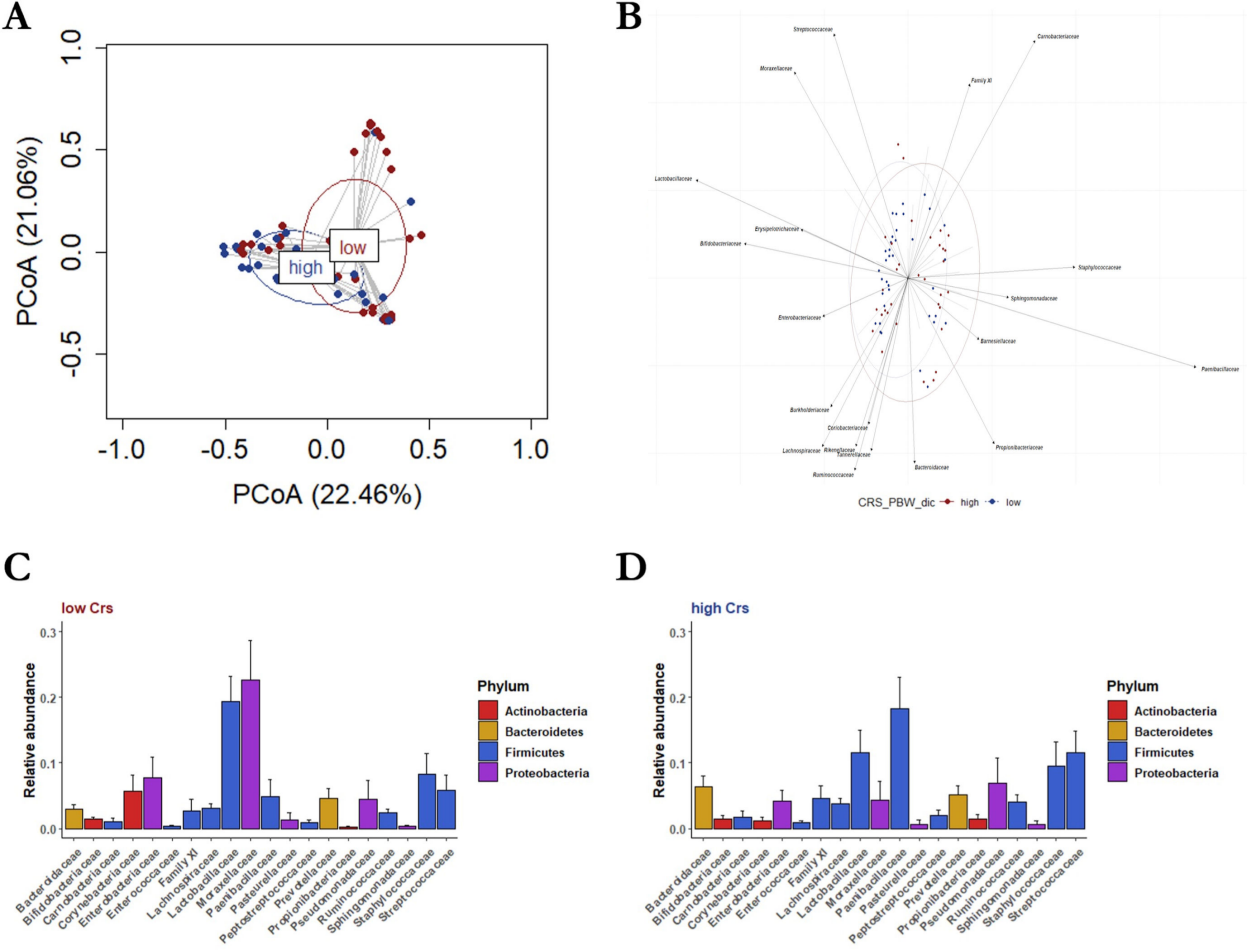
Lung microbiota composition in “low-Crs” and “high-Crs” patients. (**A**) Low-Crs patients (shown as red-colored circles and ellipse) had a bacterial community composition that differed from that of high-Crs patients (shown as blue-colored circles and ellipse), as assessed by permutational multivariate analysis of variance and visualized by principal coordinate analysis. (**B**) Biplot analysis showed the family-level bacterial taxa underlying the differences. (**C**) Rank abundance analysis of dominant family taxa revealed that the lung microbiota of low-Crs patients was enriched of Moraxellaceae and Lactobacillaceae, whereas that of (D) high-Crs patients was enriched with Paenibacillaceae, as assessed by the Kruskall–Wallis test.

**Fig 2:**
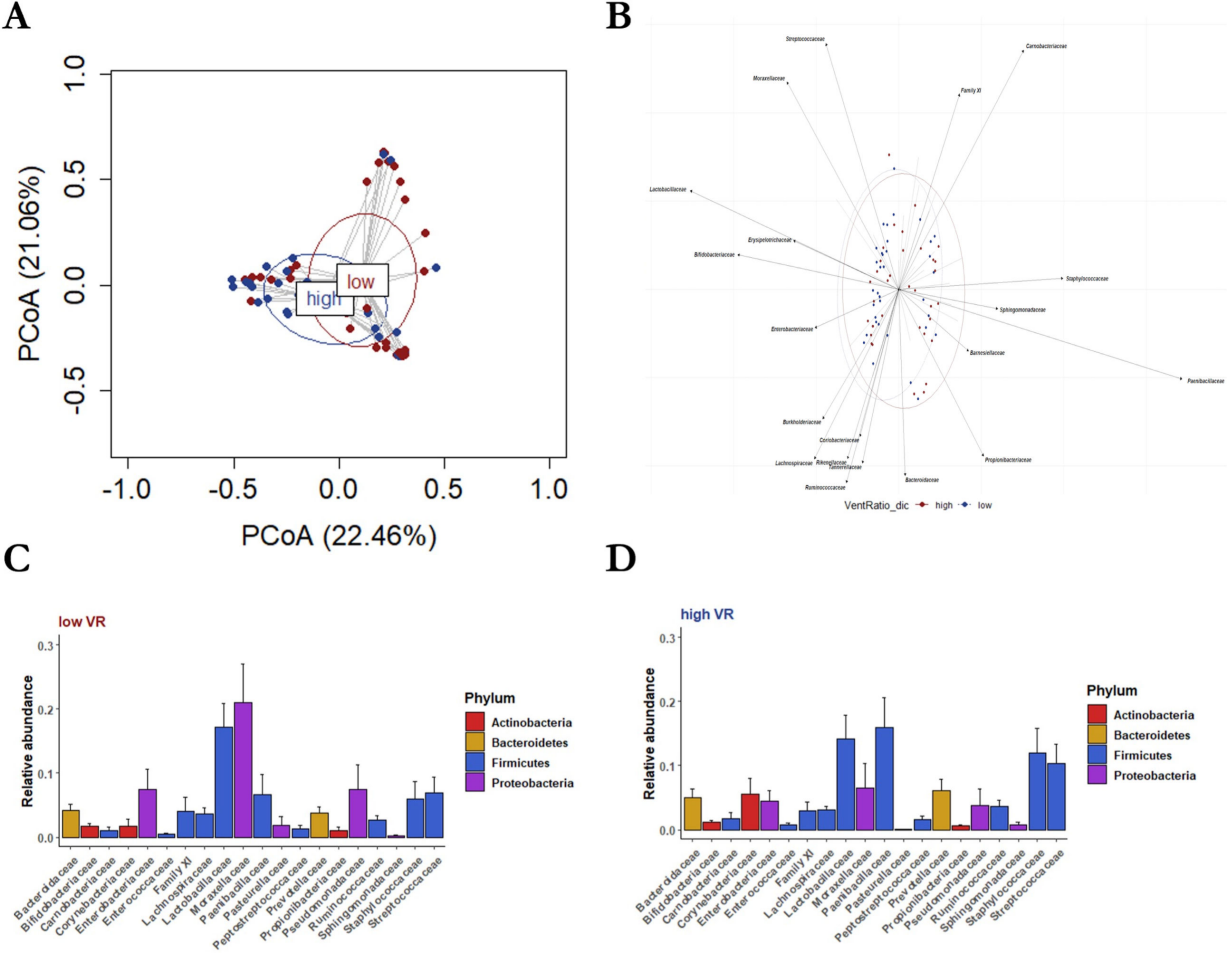
Lung microbiota composition in “low-VR” and “high-VR” patients. (**A**) Low-VR patients (shown as red-colored circles and ellipse) had a bacterial community composition that differed from that of high-VR patients (shown as blue-colored circles and ellipse), as assessed by permutational multivariate analysis of variance and visualized by principal coordinate analysis. (**B**) Biplot analysis showed the family-level bacterial taxa underlying the differences. (**C**) Rank abundance analysis of dominant family taxa revealed that Paenibacillaceae were less dominant in low-VR patients than in (D) high-VR patients, as assessed by the Kruskall–Wallis test.

**Fig 3:**
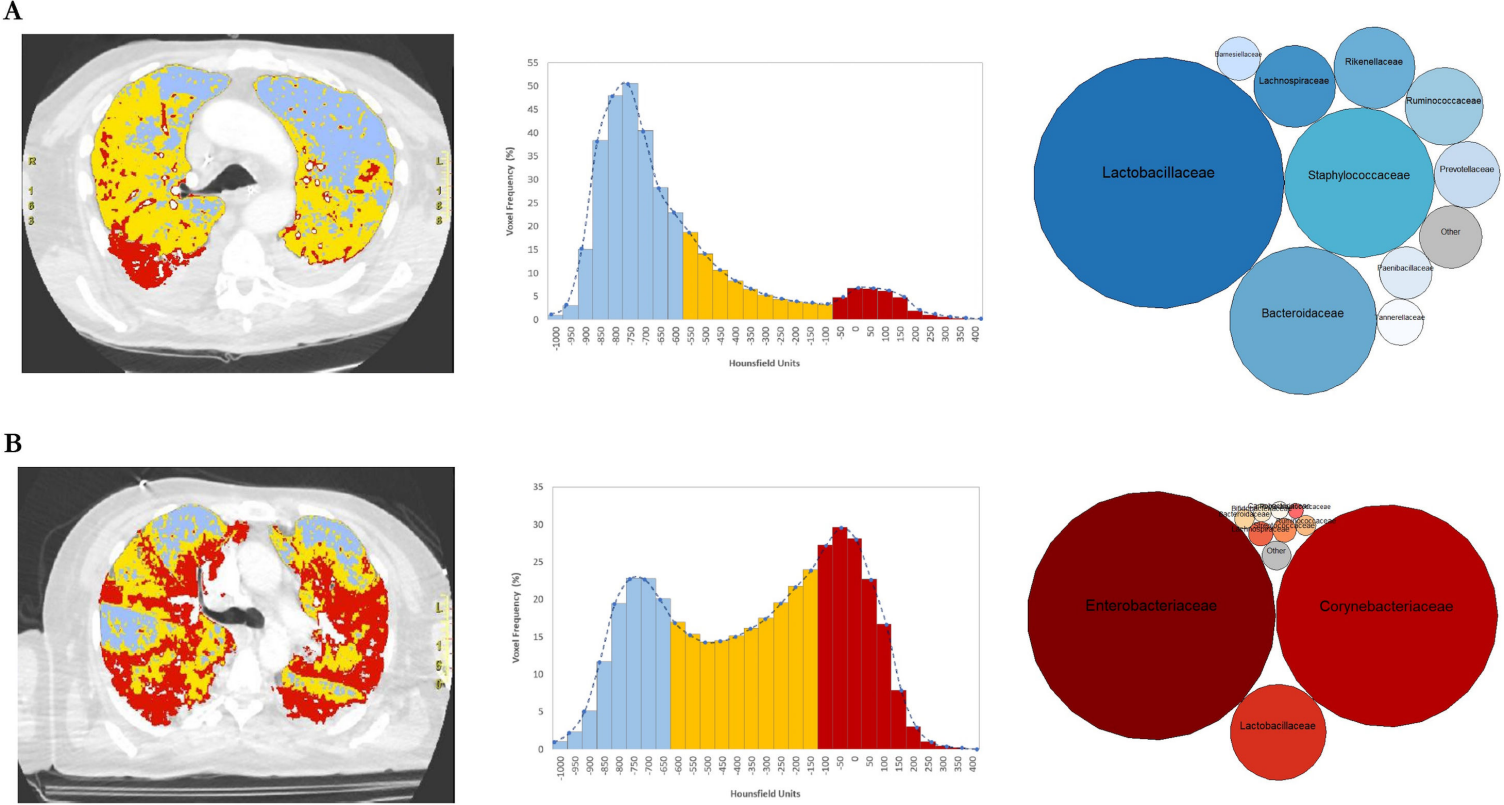
Graphic representation of respiratory mechanics, aerated tissue, and lung microbiota composition in two patients. Computed tomography scan images of lungs from two representative patients with (**A**) high Crs/PBW (0.61 mL/cmH_2_O/kg) and VR (3.64) values or (**B**) low Crs/PBW (0.43 mL/cmH_2_O/kg) and VR (1.65) values depicted the distribution of aerated lung tissue, which was detailed in the respective wave frequency graphs. For each patient, the bubble chart shows dominant families in the lung bacterial community

We compared lung bacterial communities of patients with low Crs vs high Crs and those of patients with low VR vs high VR (eFigures 4–8). In the alfa-diversity (within-sample diversity) analysis (eTable 3), communities of low-Crs or low-VR patients did not differ from those of high-Crs or high-VR counterparts when assessed both in terms of community richness (observed species per sample; *P* = 0.075 and *P* = 0.075, respectively) and Shannon diversity index (*P* = 0.256 and *P* = 0.805, respectively). In the beta-diversity (between-sample diversity) analysis, the principal coordinate analysis (PCoA)-based lung microbiota composition showed that samples from low-Crs patients overlapped with those from high-Crs patients (Fig. 1A). This was also noted for samples from low-VR patients and high-VR patients (Fig. 2A), indicating that the experimental design was appropriate. However, whereas biplot analysis allowed for visualization of dominant (i.e., observed at >0.1%) family taxa across the groups (Fig. 1B and 2B), differences [on permutational multivariate analysis of variance (PERMANOVA)] were noted at the phylum (*P* = 0.012), class (*P* = 0.015), order (*P* = 0.028), family (*P* = 0.004), and genus (*P* = 0.008) levels for the low-Crs vs high-Crs comparison and at the phylum (*P* = 0.010), class (*P* = 0.008), and order (*P* = 0.034) levels for the low-VR vs high-VR comparison (eTable 3). Moreover, relative abundance analysis (eTables 4–9) showed that Firmicutes were less dominant and Proteobacteria prevailed in low-Crs patients than in high-Crs patients (*P* = 0.017 and *P* = 0.04, respectively), while Proteobacteria were more dominant in low-VR patients than in high-VR patients (*P* = 0.013) (eTables 10–14). At the family level (Fig. 1C and 2C), Moraxellaceae and Lactobacillaceae were more dominant and Paenibacillaceae less dominant in low-Crs patients as compared to high-Crs patients (*P* = 0.040, *P* = 0.021, and *P* = 0.004, respectively), whereas Paenibacillaceae were less dominant in low-VR patients than in high-VR patients (*P* = 0.037). An example of these findings is reported in Fig. 3.

### Relationship of lung microbiota composition with C-ARDS laboratory variables

**Fig 4:**
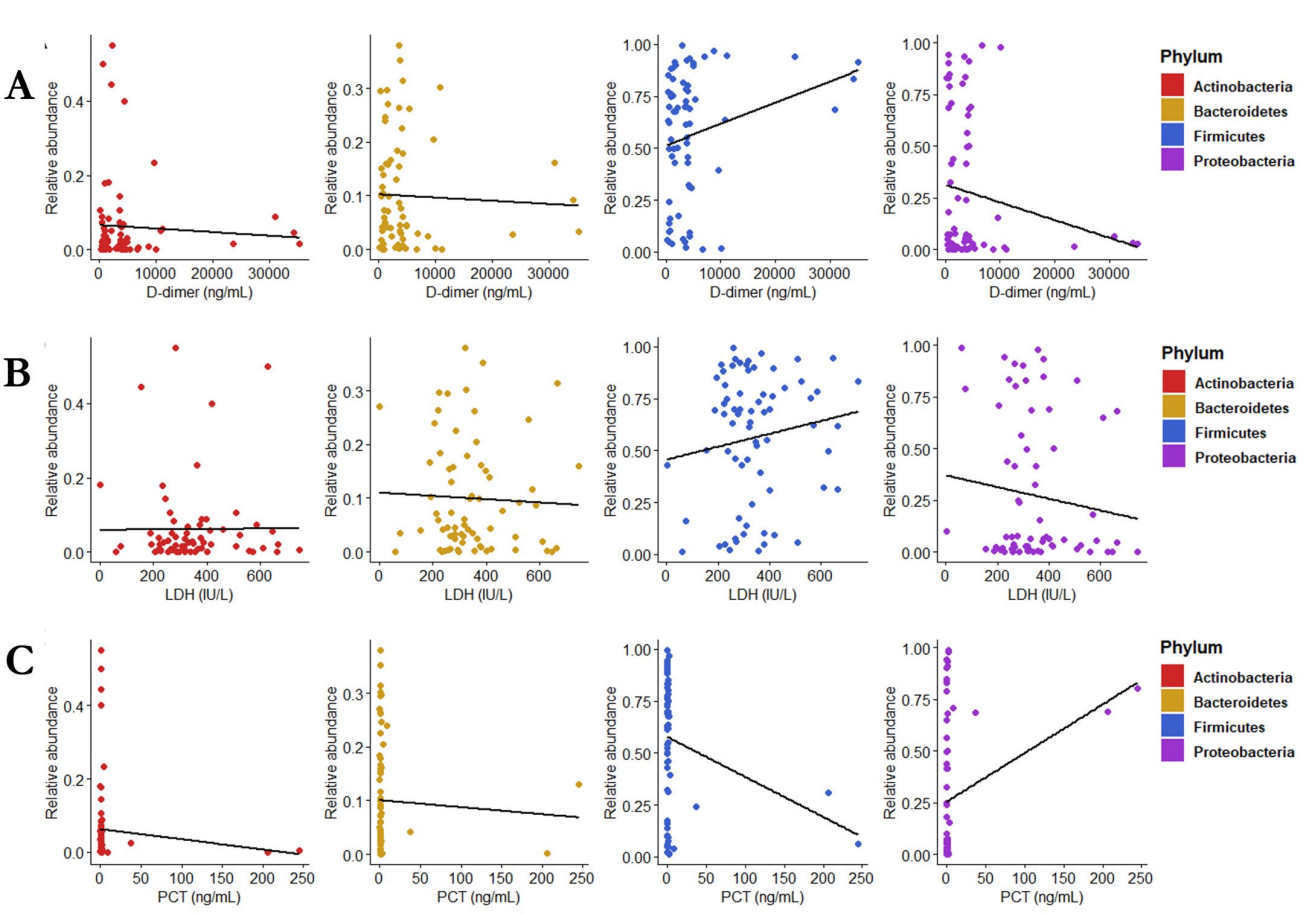
Correlation between serum procalcitonin and lung microbiota composition. Relationship assessment between lung microbiota-composing phyla and C-ARDS laboratory variables by the use of Spearman’s rank test showed the absence of correlation of Actinobacteria and Bacteroidetes with the (A) D-dimer, (B) LDH, or (C) PCT serum level. Firmicutes and Proteobacteria were, respectively, found to correlate negatively or positively only with the (C) PCT serum level.

We investigated the relationship of lung microbiota-composing phyla (including Proteobacteria and Firmicutes) with the C-ARDS laboratory variables (D-dimer, LDH, and procalcitonin) (Table 1). We observed a significantly negative correlation between Firmicutes and procalcitonin (*P* = 0.022) and a positive correlation between Proteobacteria and procalcitonin (*P* = 0.024). No correlation was instead observed for Firmicutes or Proteobacteria with D-dimer or LDH, respectively, as well as for Actinobacteria or Bacteroidetes with any of these variables (Fig. 4).

### Predictors of C-ARDS-related outcomes

**Fig 5:**
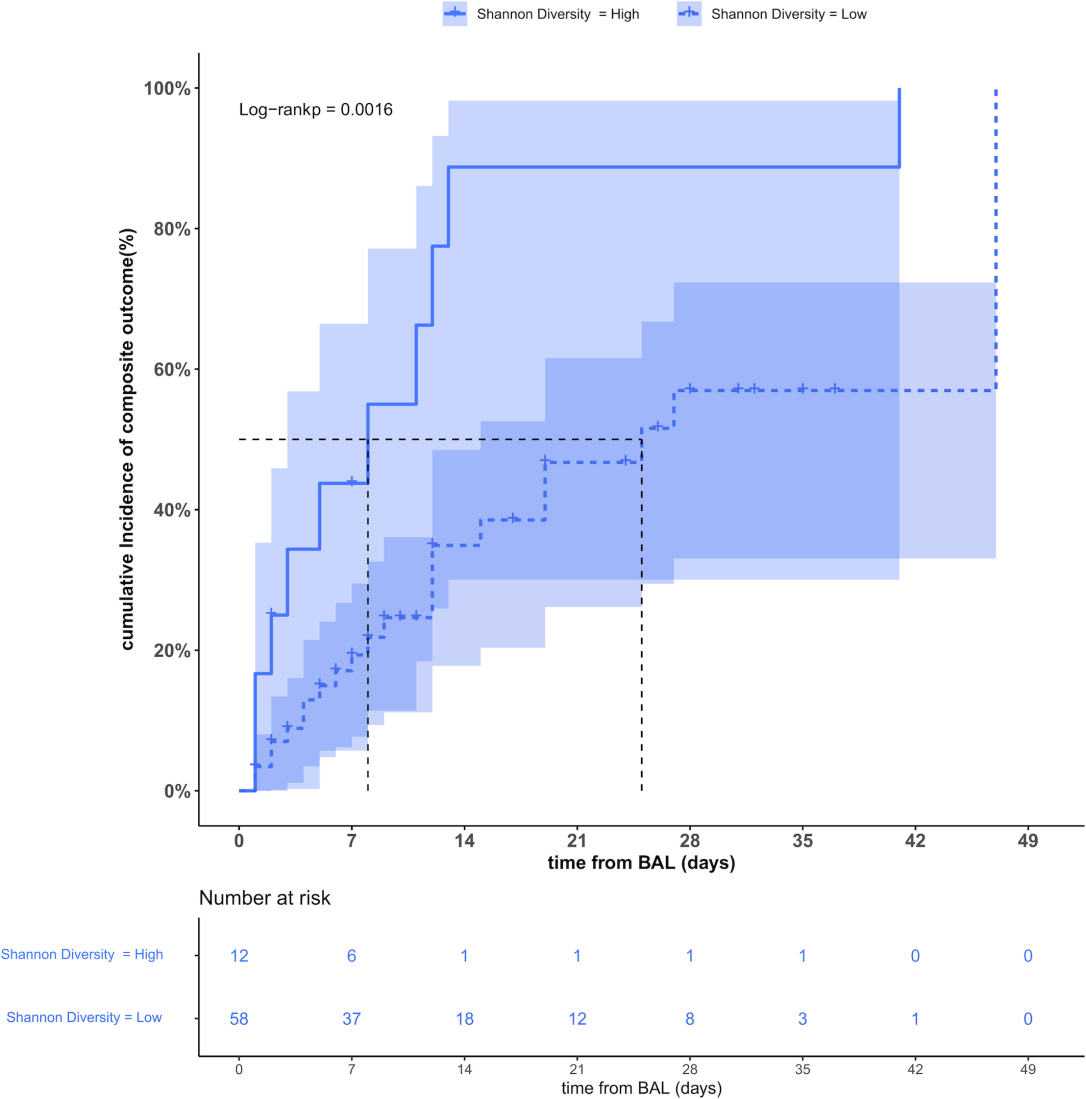
Cumulative incidence of the primary outcome according to the Shannon diversity index. Kaplan–Meier curve analysis showed that patients with a high Shannon diversity index value (solid blue line) differed significantly from patients (dotted blue line) with a low Shannon diversity value regarding the median time from BAL sampling to being weaned from mechanical ventilation and alive (8 and 25 days, respectively, log-rank *P* = 0.0016).

We finally investigated potential predictors of weaning from IMV and hospital survival (Table 2). In addition to demographic, clinical, and respiratory mechanics variables shown in Table 1, we analyzed lung microbiota diversity or composition measures. At univariable analysis, comparison of patients weaned and survived (*n* = 31) with patients not weaned from IMV and died (*n* = 39) allowed to identify the duration of symptoms before BAL sampling [hazard ratio (HR); 95% confidence interval (CI), 0.95 (0.91–0.99); *P* = 0.030] as a positive prognostic factor and the PaO_2_/FiO_2_ ratio [HR (95% CI), 1.01 (1.00–1.01); *P* = 0.002], the oxygen saturation index [HR (95% CI), 1.10 (1.05–1.15); *P* < 0.001], and the Shannon diversity index [HR (95% CI), 3.31 (1.52–7.20); *P* = 0.002] at BAL sampling as negative prognostic factors. The multivariable analysis confirmed only the Shannon diversity index [HR (95% CI), 2.35 (1.01–5.48); *P* = 0.048] as an independent predictor of negative outcome. We further assessed the cumulative incidence of the above-mentioned outcome by a KM curve analysis of patients stratified by Shannon diversity cut-off value. Of note, patients with a Shannon diversity index >2.76 (*n* = 12), when compared to patients with a Shannon diversity index ≤2.76 (*n* = 58), disclosed a significantly lower median time to being weaned from IMV and discharged alive from the hospital (8 and 25 days, respectively; *P* = 0.0016) (Fig. 5). Furthermore, no significant differences were found in the number and type of antibiotics received stratified according to low vs high Shannon diversity index [42/58 (72.4%) vs 8/12 (66.7%); *P* = 0.73]—*low diversity*: macrolides (*n* = 12), cephalosporins (*n* = 12), beta-lactam-inhibitor combination (BLIC, *n* = 8), tigecycline (*n* = 5), linezolid (*n* = 4), and quinolones (*n* = 1); *high diversity*: macrolides (*n* = 4), cephalosporines (*n* = 0), BLIC (*n* = 2), tigecycline (*n* = 0), linezolid (*n* = 1), and quinolones (*n* = 1).

**TABLE 2:** Cox proportional hazards analysis to identify predictors of in-hospital composite outcome for COVID-19 ARDS patients^*a*^

Variables	Patients who were		Univariable analysis		Multivariable analysis	
	Not weaned from MV and died (*n* = 39)	Weaned from MV and survived (*n* = 31)	HR (95% CI)	*P* value	HR (95% CI)	*P* value
Age (years)	69.6 (8.2)	64.1 (9.0)	0.99 (0.94–1.03)	0.504		
Female patients	22 (56.4)	27 (87.1)	0.36 (0.13–1.04)	0.060		
Comorbidities						
Hypertension	20 (51.3)	15 (48.4)	0.78 (0.37–1.62)	0.504		
Coronary artery disease	7 (17.9)	7 (22.6)	1.24 (0.53–2.90)	0.626		
Immunosuppression	9 (23.1)	5 (16.1)	1.19 (0.45–3.16)	0.725		
Diabetes mellitus	5 (12.8)	7 (22.6)	1.69 (0.90–3.16)	0.099		
Neoplasm	6 (15.4)	2 (6.5)	0.88 (0.21–3.70)	0.857		
Chronic heart disease	3 (7.7)	4 (12.9)	1.40 (0.49–4.03)	0.532		
Chronic obstructive pulmonary disease	5 (12.8)	1 (3.2)	0.72 (0.16–3.36)	0.681		
Chronic vasculopathy	2 (5.1)	2 (6.5)	3.69 (0.83–16.47)	0.087		
Charlson comorbidity index	3 (2–4)	2 (1–3)	0.84 (0.66–1.07)	0.160		
Ventilator-associated pneumonia	22 (56.4)	19 (61.3)	0.79 (0.37–1.66)	0.533		
SAPS II	43.1 (17.5)	36.6 (15.1)	0.99 (0.97–1.01)	0.310		
Previous hospitalization	11 (28.2)	11 (35.5)	1.31 (0.62–2.77)	0.473		
Days from the onset of symptoms	14.9 (7.8)	11.7 (8.1)	0.95 (0.91–0.99)	**0.030**	0.95 (0.91–1.00)	0.056
C-ARDS clinical/laboratory variables						
Steroid therapy receipt	21 (53.8)	11 (35.5)	0.53 (0.25–1.14)	0.103		
Interleukin-6 inhibitor receipt	4 (10.3)	6 (19.4)	1.43 (0.58–3.52)	0.433		
Antibiotics receipt^*b*^	26 (66.7)	24 (77.4)	0.58 (0.19–1.7)	0.325		
White blood cell count (10^9^/L)	15.3 (5.7)	12.9 (6.3)	0.96 (0.90–1.02)	0.192		
Lymphocyte count (10^9^/L)	1.1 (0.7–1.7)	1.0 (0.6–1.4)	0.94 (0.69–1.28)	0.697		
Neutrophil count (10^9^/L)	12.8 (6.0)	11.0 (5.8)	0.98 (0.92–1.04)	0.474		
D-dimer level (ng/mL)	3525 (1164–4135)	3554 (1029–4658)	1.00 (1.00–1.00)	0.936		
Fibrinogen level (ng/mL)	644.1 (266.2)	659.2 (240.4)	1.00 (1.00–1.00)	0.607		
Lactate dehydrogenase level (IU/L)	375.5 (156.1)	366.2 (325.9)	1.00 (1.00–1.00)	0.244		
Procalcitonin level (ng/mL)	0.54 (0.18–1.14)	0.23 (0.14–0.55)	0.71 (0.39–1.29)	0.268		
C-reactive protein level (mg/L)	150.5 (50.6–242.3)	132.4 (80.8–196.0)	1.00 (1.00–1.00)	0.338		
PaO_2_/FiO_2_ ratio (mmHg)	142.0 (42.1)	173.4 (72.6)	1.01 (1.00–1.01)	**0.002**	1.00 (0.99–1.01)	0.530
PaCO_2_ (mmHg)	50.8 (10.8)	48.5 (11.3)	1.01 (0.97–1.04)	0.732		
Oxygen saturation index	9.9 (8.8–13.6)	15.0 (11.1–17.5)	1.10 (1.05–1.15)	**<0.001**	1.07 (0.99–1.16)	0.068
Respiratory mechanics measures						
Tidal volume/PBW (mL/kg)	6.0 (5.2–6.8)	6.0 (5.4–6.7)	0.99 (0.95–1.03)	0.557		
Respiratory rate (bpm)	28.8 (3.7)	26.3 (4.9)	0.95 (0.88–1.01)	0.115		
PEEP (cmH_2_O)	10 (8–13)	11 (8–15)	1.08 (0.97–1.20)	0.153		
*P*_PLAT_ (cmH_2_O)	23.4 (3.4)	23.0 (3.8)	0.99 (0.90–1.09)	0.829		
Driving pressure (cmH_2_O)	13.1 (4.2)	11.8 (3.0)	0.92 (0.82–1.02)	0.114		
Recruitment to inflation ratio	0.50 (0.29)	0.64 (0.45)	2.04 (0.95–4.38)	0.067		
Corrected minute ventilation (L)	13.9 (11.6–16.2)	13.6 (10.2–15.8)	1.02 (0.95–1.10)	0.531		
Crs (mL/cmH_2_O)	33.6 (15.1)	37.3 (9.9)	1.02 (0.99–1.05)	0.116		
ELrs/PBW (cmH_2_O/mL/kg)	2.0 (1.6–2.5)	1.8 (1.6–2.2)	0.64 (0.37–1.09)	0.102		
Crs/PBW [(mL/cmH_2_O/kg (low value)]*^c^*	15 (38.5)	18 (58.1)	0.49 (0.23–1.04)	0.063		
VR (high value)*^d^*	21 (53.8)	13 (41.9)	0.86 (0.42–1.78)	0.690		
Lung microbiota features						
Observed species	70.0 (28.0–96.5)	66.0 (16.0–86.5)	1.00 (0.99–1.01)	0.511		
Shannon diversity index (high value)*^e^*	2 (5.1)	10 (32.3)	3.31 (1.52–7.20)	**0.002**	2.35 (1.01–5.48)	**0.048**
Phylum taxa (relative abundance)						
Actinobacteria	0.02 (0.01–0.07)	0.02 (0.00–0.04)	0.07 (0.00–12.27)	0.320		
Bacteroidetes	0.02 (0.00–0.05)	0.02 (0.00–0.09)	25.69 (0.63–104.0)	0.086		
Firmicutes	0.64 (0.17–0.78)	0.69 (0.44–0.89)	2.82 (0.87–9.13)	0.083		
Proteobacteria	0.05 (0.00–0.69)	0.06 (0.02–0.42)	0.45 (0.15–1.31)	0.143		

^*a*^ Data are expressed as absolute and relative percentage frequency for qualitative variables, while either mean and SD or median and interquartile range (IQR) were applied to quantitative variables, as appropriate. Bold indicates statistical significance, which was set at a *P* < 0.05. Only statistically significant variables in the univariable analysis were entered into the multivariable model, due to the low number of events per variable. PBW, predicted body weight; SAPS, simplified acute physiology score; MV, mechanical ventilation; C-ARDS, COVID-19-related ARDS; PaO_2_/FiO_2_, partial pressure of arterial oxygen/fraction of inspired oxygen; RR, respiratory rate; PaCO_2_, arterial partial pressure of carbon dioxide; PEEP, positive end-expiratory pressure; *P*_PLAT_, plateau pressure; Crs, compliance of the respiratory system; ELrs, elastance of the respiratory system; VR, ventilatory ratio.

^*b*^ Not weaned from MV and died: macrolides (*n* = 6), cephalosporins (*n* = 6), beta-lactam-inhibitor combination (BLIC*, n* = 7), tigecycline (*n* = 4), linezolid (*n* = 3), and quinolones (*n* = 0). Weaned from MV and alive: macrolides (*n* = 10), cephalosporins (*n* = 6), BLIC (*n* = 3), tigecycline (*n* = 1), linezolid (*n* = 2), and quinolones (*n* = 2).

^*c*^ Low value refers to as the mean value across the patient cohort (*n* = 70), which was used to stratify patients as low-Crs (Crs/PBW values, ≤0.53) or high-Crs (Crs/PBW values, >0.53) patients. The high value was set as the reference.

^*d*^ High value refers to as the median value across the patient cohort (*n* = 70), which was used to stratify patients as low-VR (VR values, ≤2.2) or high-VR (VR values, >2.2) patients. The low value was set as the reference value.

^*e*^ Versus a low (reference) value, which was calculated with respect to the median value (2.76) across the patient cohort (*n* = 70). Accordingly, patients’ lung microbiota was categorized as having low (≤2.76, 58 patients) or high (>2.76, 12 patients) Shannon diversity index.

## DISCUSSION

We observed that BAL-isolated bacterial communities were associated with Crs and VR. Moreover, we identified one community dominated by Proteobacteria (with the archetypal *Acinetobacter*) and another dominated by Firmicutes (with the archetypal *Lactobacillus* or *Paenibacillus*), regardless of respiratory mechanics alterations. Also, we found that Proteobacteria positively correlated, while Firmicutes negatively, with serum procalcitonin, suggesting that Proteobacteria may contribute to ARDS-related systemic inflammation. Furthermore, patients with greater lung microbiota diversity had a significantly higher chance of being weaned from IMV and discharged alive from the hospital.

Consistent with the concept that oxygenation and ventilation are physiologically distinct processes (5), Crs and VR values allowed the identification of patients with different lung diseases, thus reflecting a different microbiota from healthy human lungs (14). It is likely that physicochemical (oxygen tension or pH) variations of the lung favored the predominance of Proteobacteria and Firmicutes over other species (14). The predicted lower number of aerated lung units in low-Crs patients as compared to high-Crs patients may have stimulated a bloom of facultative anaerobic Proteobacteria (mainly Gammaproteobacteria) (3). These include Moraxellaceae species commonly resident in the human oral cavity (15), but that, whenever dispersed to the lungs (9), may be actively selected by injured alveoli (6). Firmicutes such as Lactobacillaceae species usually maintain an acidic niche in the lung, whereas Proteobacteria—including *Acinetobacter* or *Pseudomonas* species—may be hindered from thriving (16). However, the Firmicutes/Proteobacteria ratio can reverse due to an unstable structure of the lung microbiota, which may also be affected by abnormal CO_2_ metabolism during C-ARDS. Increased levels of PaCO_2_ might explain the lesser dominance of Firmicutes of high-VR patients as compared to low-VR patients.

Although MV is the mainstay of ARDS management (17, 18), it may be *per se* harmful (3). Due to this reason, a lung-protective ventilation strategy has become the standard of care (2) to minimize the occurrence of ventilator-induced lung injury (19). At BAL sampling, patients had been ventilated for at least 2 days, a time during which C-ARDS-related changes in the lung microbiota may have created favorable conditions for Ventilator-Associated Pneumonia (VAP) development (20). Although extensive clinical overlap among ARDS, pneumonia, and COVID-19 makes it difficult to establish the contribution of VAP to the risk of death in the ICU, it is essential to rely on the prevention of VAP on the early weaning from IMV (21).

The association of Crs and VR with poor outcomes in COVID-19 patients either with (11) or without (22) established ARDS has already been shown. Therefore, we investigated whether distinct lung microbiota features could have been associated with the risk of not being weaned from IMV and death. After controlling for potential predictors of death, including VAP, we stratified the outcome by the Shannon diversity index, which represents the most accurate way to measure species diversity in the lung bacterial community (20). In one study on BAL fluid samples from 91 patients (17 with and 74 without ARDS) (12), the overall lung community composition (which was enriched of gut-associated taxa such as Lachnospiraceae and Enterobacteriaceae) (23), but not the Shannon diversity index was significantly predictive of worse ICU outcomes (i.e., extubation and 28-day survival). Other indices of lung bacterial diversity were not significantly predictive of ICU outcomes as in our study, whereas the discrepancy in the Shannon diversity index between that study (12) and ours could be due to the inherently different patient cohorts studied, with our cohort including only ARDS patients. This relative homogeneity in our patient population provided the right background from which differences in the lung microbiota structure could be determined. Unfortunately, we did not provide support for the hypothesis of lung bacterial taxa abundance contributing to clinical outcomes in C-ARDS patients.

To make consistent C-ARDS lung microbiota findings, putative contaminant 16S RNA gene sequences in BAL fluid samples were removed from the analysis. This was consistent with previously developed strategies to limit the negative impact of DNA contamination that arises especially when low microbial biomass samples are analyzed (24). Unsurprisingly, three clusters of taxa, such as pharyngeal-associated taxa, inflammation-associated taxa, or contamination-associated taxa, were identified in the lung bacterial community of healthy lung transplant recipient patients (25). Similarly, the removal of contaminant DNA sequences allowed us to virtually enrich BAL fluid samples with taxa that originated through oral-cavity immigration (i.e., Firmicutes), gut–lung translocation (i.e., Proteobacteria), or aspiration of an altered pharyngeal microbiota.

Finally, it is noteworthy that non-COVID-19 ARDS may present similar aspects in terms of dysbiosis evidence (low alpha diversity and low relative abundance of “protective” oral origin commensal bacteria), especially for the phenotypes with reduced Crs rather than increased VR (12, 23, 26). Indeed, in both animal and human studies before the COVID-19 pandemic, an enrichment of the lung microbiome with gut bacteria and a decrease in alpha diversity were associated with the occurrence of ARDS, either primary or sepsis-induced (6). Conversely, Kullberg and coworkers found that, in COVID-19 patients, the lung microbiota community composition, linked to total bacterial burden rather than to a- and b-diversity, was associated with successful extubation (13). Unfortunately, we did not investigate the bacterial burden of our patients, but in light of the observed results, we can conclude that both papers support the role of lung microbiota as a driver of non-resolving ARDS and of the complex heterogeneity of such disease.

Our study has some strengths. First, it is one of the few studies investigating the clinical implications of lung microbiota composition in a relatively large number of patients. Second, this is the first study investigating the relationship between lung microbiota features and respiratory mechanics in patients with C-ARDS. However, this study has some limitations. First, we have only focused on single Crs or VR measurements, not considering the clinical evolution at different time points. The ICU admission before intubation was longer for some patients, which may lead to a potential bias associated with different C-ARDS stages and respiratory mechanics evaluation. Second, we assumed that the lung microbiota characteristics of our patients were typical of C-ARDS, as we did not include ARDS patients without COVID-19. Third, we correlated lung microbiota characteristics with classical biomarkers of cell damage, micro-thrombosis, or infection, whereas no potential relationships with other markers of inflammation were explored. In addition, our lung microbiota characterization targeted only the bacterial component, so we cannot draw any conclusion regarding the role of residual viral replication as a contributing factor to the observed VR/Crs differences and final outcome. While this should improve the comparability of our findings with those of most published studies, we ignored viral or fungal components of the resident microbial community in the lungs. Appreciating these components would have allowed to obtain important information about the abundances (and their clinical significance) of SARS-CoV-2 or other viruses as well as those of *Candida* or *Aspergillus* organisms in the lung microbiota of our patients. Future studies are needed to fill the inevitable gap in such a complex and intricate subject of investigation.

### Conclusions

Lung bacterial communities of mechanically ventilated C-ARDS patients differed based on the dominance of bacterial taxa, and the observed differences were related to patients’ respiratory mechanics. C-ARDS patients with low Crs/low VR had a Proteobacteria-dominated lung microbiota; conversely, Firmicutes were dominant in the high Crs phenotype. Furthermore, loss in microbiota diversity appeared to be associated with a longer duration of IMV and higher mortality. Early patient stratification driven by microbiota diversity may help to deliver target intervention, thus aiming to optimize the management C-ARDS patients.

## MATERIALS AND METHODS

### Study design and patient characteristics

This prospective observational study was performed in two medical ICUs of a tertiary university hospital in Italy from 1 April through 31 December 2020. The study included adult (≥18 years) critically ill patients with COVID-19 ARDS requiring IMV consecutively admitted to the ICU, who matched the following criteria: (i) execution of BAL sampling and (ii) acquisition of the informed consent. Patients were excluded if one of the following conditions was verified: (i) clinical contraindications to BAL sampling (severe hypoxemia, barotrauma, and coagulation abnormalities); (ii) clearly bloody BAL sample and/or small quantity of BAL sample (<5 mL) impairing the 16S ribosomal RNA (rRNA) processing for microbiota analysis (Supplemental material) (Fig. S1 for flow chart of the study). The study was performed in accordance with the Declaration of Helsinki and was approved by the Ethics Committee of Fondazione Policlinico Agostino Gemelli IRCCS (reference number 23703/19). This study was funded by the Italian Ministry of Health (“Lung Microbiota Analysis in Critically ill Patients Admitted to the Intensive Care Unit”—NCT04271345). Written informed consent or proxy consent was obtained before the inclusion in the study. This manuscript was written in compliance with the " The Strengthening the Reporting of Observational Studies in Epidemiology" (STROBE) guidelines. An extensive description of demographic, clinical, and laboratory data was provided in the Supplemental material. Data were prospectively collected via an electronic database, which was stored in an Excel spreadsheet and shared by investigators.

### Main outcomes

The primary outcome of this study was the relationship of lung microbiota (diversity and community composition) with respiratory mechanics assessed by VR and Crs (Crs/predicted body weight; hereinafter referred to as Crs).

Secondary outcomes included the identification of potential predictors of successful weaning from IMV and survival at hospital discharge, which were analyzed as a single composite outcome (Supplemental material).

### Sample processing and 16s rRNA gene sequencing

BAL fluid samples were stored at −80°C until processing via 16S rRNA gene sequencing (Supplemental material). Briefly, a 5 mL aliquot was centrifuged, the pellet was suspended into sterile phosphate buffer, and the suspension was used for DNA extraction using the DANAGENE MICROBIOME Saliva DNA kit (DanaGen-BioTed S.L., Barcelona, Spain). High-quality DNA were underwent PCR amplification of 16S rRNA gene V3–V4 hypervariable regions using primers and conditions as previously described (27). Purified amplicons were used to generate DNA libraries, which were submitted to sequencing on the Illumina MiSeq instrument (Illumina, San Diego, CA, USA). The raw sequencing reads were processed as described below. To minimize the impact of microbial contaminant DNA in low-biomass samples such as BAL fluid (24), additional samples (i.e., extraction blank controls) were processed and sequenced alongside the study samples.

### Bioinformatics and statistical analyses

Sequencing reads were trimmed, quality-filtered, and merged using the QIIME2 pipeline (v2020.06) (Supplemental material). Taxonomic assignment of each representative amplicon sequence variant was performed by VSEARCH against the SILVA 132 16S rRNA database (28). Bacterial community analysis was performed with R studio v4.0.2 using the *phyloseq* software package (29), whereas potential contaminant sequences were assessed using the *decontam* R package (30). Prior to diversity and relative abundance analyses, sequences from taxa not observed two times or more in at least 5% of samples were excluded. This resulted in 3,866,753 total reads (median value, 49,791 reads) corresponding to 447 taxa, whereas the sequences’ data set was normalized to 49,791 reads per sample. Alpha diversity (i.e., the within-sample diversity) was measured using the observed species and Shannon diversity index, whereas beta diversity (i.e., the between-sample diversity) by the Bray–Curtis distance and visualized by PCoA. Statistical differences between groups were identified using the Kruskal–Wallis test for alpha diversity or the PERMANOVA for beta diversity. Relative abundances were calculated at any taxonomic level (e.g., phylum), and statistical significance between groups was assessed by the Kruskal–Wallis test.

Clinical data analysis was performed using R software v4.2.0 (R Core Team, 2022; Wien, Austria) (Supplemental material). Qualitative data were expressed as absolute and relative percentage frequency, whereas quantitative data were expressed as either mean and SD or median and interquartile range (IQR). Gaussian distribution was previously assessed by the Shapiro–Wilk’s test. Differences between groups were assessed by *χ^2^* test or Fisher–Freeman–Halton’s exact test for qualitative data, whereas either Student’s *t* test or Mann–Whitney *U* test was applied to quantitative data, as appropriate. Correlation analysis was performed by Spearman’s rank correlation test. Both uni- and multivariable Cox regression models were fitted to assess predictors of successful weaning from IMV and hospital survival (composite outcome). HRs and 95% CIs were reported. KM survival analysis was applied to assess potential differences in the composite outcome between specific groups. Log-rank *P* and KM curve were further performed. Due to the complete exploratory nature of the study, we could not calculate a sample size, but for the multivariable Cox regression model, we followed the rule to include 1 predictor every 10 events. For the whole analyses, statistical significance was set at a *P* value <0.05.

## Data Availability

The datasets used and/or analyzed during the current study are available from the corresponding author on reasonable request

## SUPPLEMENTAL MATERIAL

ESM spectrum.03574-23-s0001.docx Supplemental material.

ASM does not own the copyrights to Supplemental Material that may be linked to, or accessed through, an article. The authors have granted ASM a non-exclusive, world-wide license to publish the Supplemental Material files. Please contact the corresponding author directly for reuse.
