# Supplementary material for: Lung microbiota composition, respiratory mechanics, and outcomes in COVID-19-related ARDS
Source: Microbiol Spectr. 2024 Mar 11;12(4):e03574-23. doi: 10.1128/spectrum.03574-23 (PMC10986322; doi:10.1128/spectrum.03574-23)
Supplement: ESM — Supplemental material. [file spectrum.03574-23-s0001.docx]

**Experimental Materials and Methods**

*Study patients, samples, and outcomes*

This study included patients (n=70) who received invasive mechanical ventilation (MV) through endotracheal tube at the intensive care unit (ICU) of Policlinico Universitario Agostino Gemelli IRCCS of Rome (Italy) for COVID-19-related acute respiratory distress syndrome (ARDS). At the time of bronchoalveolar lavage (BAL) sampling (see below), patients were diagnosed with ARDS based on the Berlin definition,^1^ and had a severe acute respiratory syndrome coronavirus 2 (SARS-CoV-2) infection (i.e., the notorious cause of COVID-19), which had been laboratory confirmed upon ICU admission via nasopharyngeal swab reverse transcription (RT)-PCR assay.^2^ Exclusion criteria for eligible patients (n=230), as specified in the study selection flow diagram (see **eFigure 1**), referred to 134 patients with BAL sampling-incompatible clinical conditions, 25 patients with BAL fluid samples inadequate for 16S rRNA sequencing analysis (see below), or 1 patient with a BAL fluid sample which provided insufficient 16S rRNA sequence reads (see below). Ultimately, 70 BAL fluid samples were included in the study.

We performed miniature BAL sampling by a double-lumen catheter (BAL Cath, Ballard Medical Products, Draper, Utah, USA), which was introduced into each patient’s airways through an endotracheal tube, so that the curved tip of the catheter was directed towards either the left or right lung, as preferred.^3^ Briefly, the outer catheter was inserted into the left or right main bronchus, as appropriate, and the inner catheter was inserted in a “wedge” position. Through the catheter, 20 mL of 0.9% saline was rapidly injected and aspirated from patient’s distal airways, after which the catheter was removed. The BAL fluid sample was immediately sent to the microbiology laboratory of above-mentioned hospital. The sample was divided into aliquots, which were kept on ice (for 60 minutes at maximum) until processing. One aliquot was submitted to microbiological investigations, consisting of Gram staining examination and (qualitative or quantitative) aerobic cultures on standard agar media.^3^ For microbial isolates, species identification was performed using the MALDI Biotyper system (Bruker Daltonics, Bremen, Germany), whilst *in vitro* antimicrobial susceptibility testing was performed using Vitek 2 (bioMérieux, Mercy l’Étoile, France) or MERLIN Diagnostica GmbH (Bornheim, Germany) broth microdilution systems. Minimum inhibitory concentrations were interpreted according to the European Committee on Antimicrobial Susceptibility Testing (EUCAST) clinical breakpoints. Surveillance cultures were performed to detect methicillin-resistant *Staphylococcus aureus* (MRSA) organisms in nasal swabs, or carbapenem-resistant *Enterobacterales* (CRE) organisms in rectal swabs. Alongside, either blood or urine cultures were obtained to detect bloodstream infection (BSI) and urine tract infection (UTI), respectively. Ventilator-associated pneumonia (VAP) was diagnosed according to previously reported criteria,^4^ which included the presence of radiological signs (i.e., a new or persistent infiltrate on chest X-ray) together with two of three following criteria: (i) purulent tracheal aspirate, (ii) hyperthermia (T >38°C) or hypothermia (T <36°C), and (iii) peripheral leukocytosis (i.e., white blood cell count of >10 × 10^9^/L or <1.5 × 10^9^/L), and when a BAL fluid culture yielded bacterial growth at or above 10^4^ colony-forming unit (CFU)/mL.^5^ All cases developed in ICU patients who required MV for at least 48 hours, thus meeting the definition of VAP.^5^

For each patient, MV was applied in a volume-controlled mode with a heat and moisture exchanger: tidal volume, 6 mL/kg of predicted body weight (PBW); inspiratory flow, 60 L/min; inspiratory pause, 0.3 sec; respiratory rate, <35 breaths/min at a pH of >7.3; fraction of inspired oxygen (FiO_2_), titrated to achieve oxygen saturation (SpO_2_) of 90–96%. Oxygenation was quantified as a ratio of the partial pressure of arterial oxygen (PaO_2_) to the fraction of inspired oxygen (FiO_2_). Respiratory mechanics measurements were conducted in supine semi-recumbent position within 24h from intubation and before any prone-positioning session.

Airway plateau pressure (Pplat) and positive end-expiratory pressure (PEEP) were measured and used, alongside the tidal volume, to calculate compliance of the respiratory system (Crs), as follows: $\left[ tidal volume \left( mL \right) \right]/\left[ \left( Pplat-PEEP \left( cmH2O \right) \right) \right]/PBW$. Elastance of the respiratory system (ELrs), which is the reciprocal of Crs,^6^ was reported in cmH_2_O/mL/kg of PBW. Driving pressure (cmH_2_O), the recruitment to inflation ratio, the corrected minute ventilation (L), and the arterial partial pressure of carbon dioxide (PaCO_2_) were also measured.

The ventilatory ratio (VR), which is a surrogate for dead space (defined as the portion of tidal volume that does not take part in gas exchange^7^), was calculated as follows: $[minute ventilation (mL/min) \times PaCO2 (mmHg)]/[PBW (kg) \times100 \times37.5]$.^8^

Patient monitoring/caring implied: (i) no routine change of ventilator circuits; (ii) tracheal suction by an open system, when necessary; (iii) periodic check every 4 hours of semi-recumbent body position to keep an angle of 30°; (iv) nasogastric tube; (v) continuous enteric nutrition; (vi) prophylaxis for stress ulcer; (vii) oral cleaning with chlorhexidine, every 8 hours, (viii) protocol of sedation, (ix) protocol for weaning from MV; and (x) no selective digestive decontamination. All patients underwent to continuous endotracheal tube cuff-pressure control and aspiration of subglottic secretions.^5^ All patients were sedated and paralyzed with continuous infusion of cisatracurium standard dose (35 mg/h).

Data collected from the study patients (see **Table 1**) included demographics (age and sex), comorbidities (including Charlson comorbidity index), simplified acute physiology score II (SAPS II), previous hospitalization, days from the onset of symptoms, days from the start of MV, C-ARDS clinical or laboratory findings, respiratory mechanics (including all above-mentioned) measures, outcome (including weaning from MV or in-hospital mortality) measures. Additional data (see **eTable 1**) included: (i) BAL fluid sample culture results; (ii) MRSA colonization status; (iii) CRE colonization status; (iv) VAP, UTI, or BSI diagnoses; and (v) empirical/therapeutic administration of antimicrobial agents.

*16S ribosomal RNA gene sequencing*

An aliquot of BAL fluid sample from each patient eligible for the study (see above) was processed for lung microbiota analysis, which began with the assessment of sample quality and volume upon receipt by the clinical microbiology technician. As already mentioned, samples were rejected if they were like saliva (i.e., either hyaline or watery with bubbles) or if they had a less than 5 mL volume. After inclusion in the study (n=71), BAL fluid samples were handled in a biosafety level (BSL)-3 laboratory. Samples were filtered to remove non-cellular material and/or mucus, then cells were separated via centrifugation (1000 g for 4 min), and the resulting supernatant was collected and stored at −80°C until 16S ribosomal RNA (rRNA) gene sequencing. To this end, each sample’s supernatant (5 mL of volume) was centrifuged (23,000 g for 20 min), and the resulting pellet was resuspended in phosphate-buffered saline (PBS) and used for microbial DNA isolation, which was performed in a strictly controlled, separate, and sterile workplace, through the DANAGENE MICROBIOME Saliva DNA kit (Danagen-Bioted, S.L., Barcelona, Spain), as previously described.^9^ We know that the kit has been developed to study the oral cavity microbiota, which is the second most abundant human microbiota after that in the gastrointestinal tract.^10^ We also know that the lungs, at least in healthy individuals, are constantly bombarded by bacteria immigrated from the oral cavity,^11^ which remains the most significant source of bacteria residing in the lungs during health.^12^ These observations supported our choice to use DANAGENE MICROBIOME Saliva DNA kit on BAL fluid samples in the previous,^9^ or present study. The extracted DNA was dissolved in DNA-free Tris buffer and was available for subsequent reactions. Before sequencing, DNA solution concentration was determined through measurement with a Qubit 4.0 fluorometer (Thermo Fisher Scientific, Rodano, Italy) and the Qubit dsDNA HS (High Sensitivity) Assay kit (Life Technologies, Monza, Italy). In each experimental run (including PCR sequencing), DNA extraction blank controls (i.e., sterile water samples) were included, as recommended,^13^ to check for contaminant DNA that has so far been shown to come from many sources during the sample processing steps in the clinical microbiology laboratory.

PCR primers were used to amplify V3–V4 hypervariable regions from the 16S rRNA gene (V3_Next_For, 5’-TCGTCGGCAGCGTCAGATGTGTATAAGAGACAGCCTACGGGNGGCWGCAG-3’; and V4_Next_Rev, 5’-TCTCGTGGGCTCGGAGATGTGTATAAGAGACAGGACTACHVGGGTATCTAATCC-3’),^9^ which had been designed to contain (from 5’ to 3’), in the order, sequences for the Nextera transposon and for BV5 (Next For) and AV6 (Next Rev) priming.^14^ Extracted DNA (5 µL at 0.5-µg/mL concentration) was used as the template in a 50-µL PCR volume, which contained 1U Phusion High-Fidelity DNA polymerase (Thermo Fisher Scientific), 1X High-Fidelity buffer (Thermo Fisher Scientific), 200-μM dNTPs, and 0.3-μM each primer.

Thermal cycling conditions were set as follows: (i) 98°C for 2 min; (ii) 20 cycles, each consisting of 98°C for 10 sec, 58°C for 30 sec, and 72°C for 15 sec; (iii) 15 cycles, each consisting of 98°C for 10 sec, 62°C for 30 sec, and 72°C for 15 sec; and (iv) 72°C for 7 min. Amplicons were purified using Agencourt AMPure XP beads (Beckman Coulter, Milan, Italy) and were eluted in 35-µL nuclease-free water. Amplicons were then checked for quality on 1% agarose gel electrophoresis, and DNA concentration was determined using the above-mentioned method. To incorporate unique Nextera XT i5 and i7 indexes to both amplicon ends, we used 20 µL (at 20-ng/ml concentration) of purified amplicons as the template in a 50-µL PCR volume, which contained 1U Phusion High-Fidelity DNA polymerase, 1X High-Fidelity buffer, 100-μM dNTPs, and 5-μL each of i5 and i7 indexes. Thermal cycling conditions were set as follows: (i) 98°C for 30 sec; (ii) 5 cycles, each cycle consisting of 98°C for 10 sec, 63°C for 30 sec, and 72°C for 3 min. Indexed amplicons were purified using Agencourt AMPure XP beads and eluted in 25-µL nuclease-free water, and amplicon quality and concentration was assessed as already described. Each sample’s indexed amplicons were equimolarly diluted, and the final amplicon pool was subjected to 2 × 300 paired-end sequencing (Illumina, San Diego, CA, USA) onto an Illumina MiSeq instrument. To increase the base-diversity degree, an internal control (PhiX v3; Illumina) was added to the DNA library.^15^

The resulting raw sequencing data were processed with QIIME2 plugins v2020.6.^16^ Demultiplexing, and quality inspection of paired-end reads were performed using the “demux” plugin, while trimming of Illumina adapter sequence (5’-CTGTCTCTTATACACATCT-3’) was performed using the “cutadapt trim-paired” plugin. Denoising of paired-end reads was performed using the “dada2 denoised-paired” plugin, which allowed to adjust the number of 5’- and 3’-end trimmed bases to remove primer sequences or low-quality sequences.^17^ This led to an approximately 70% good-merged reads output. Amplicon sequence variants (ASVs) were assembled using the “feature-table summarize” plugin, while we applied the “feature-classifier” plugin to classify ASVs at the taxonomic level by the VSEARCH global consensus alignment and the SILVA 132 16S rRNA database (at a 99% sequence similarity threshold).^18^ Sequences are available through the NCBI Sequence Read Archive (accession number PRJNA693784 and PRJNA912734). ASV, taxonomy, and metadata tables are available upon reasonable request to the corresponding author.

*Bioinformatics and statistics analyses*

Lung microbiota data analysis was performed using R v4.0.2 (https://www.rstudio.com/) and the *phyloseq* R package.^19^ First, we identified (and removed) contaminant DNA sequences via *decontam* R package v1.6,^20^ which relies on the assumption that contaminant ASVs appear at higher frequencies in low-biomass samples (frequency-based identification) and that ASVs are found in negative controls (prevalence-based identification). Next, we removed ASVs (10 in total) for which a bacterial taxonomic assignment could not be achieved (i.e., unassigned ASVs), one sample for which less than 1000 reads were obtained, and bacterial taxa not observed more than two times in at least 5% of BAL fluid samples. We thus obtained 3.908.676 reads (median value, 50.888 reads), for a total of 455 bacterial taxa. After applying an additional taxonomic filter to remove low-prevalence taxa (Epsilonbacteraeota, Patescibacteria, Tenericutes, and Verrucomicrobia), the final lung microbiota dataset included 3.866.753 reads (median value, 49.791 reads) accounting for 447 ASVs/taxa in total.

To minimize the effect of sequencing depth variation across BAL fluid samples, as assessed by rarefaction curve analysis (see **Supplementary Figure 2**), samples were normalized to 49.791 reads, which were the basis to perform downstream analyses. To measure the diversity within each BAL fluid sample (alpha diversity), we u calculated the observed species and Shannon index, respectively, whilst to measure the diversity between BAL fluid samples (beta diversity), we generated Bray-Curtis distance matrices and visualized them by principal coordinate analysis (PCoA). Additionally, we used principal component analysis on cantered log-ratio transformed normalized ASV tables to perform ordinations produced by Bray-Curtis distance matrices, graphically represented as a biplot. Statistical significance of lung microbiota alfa-diversity or beta-diversity differences between *a priori* defined groups (see below) was, respectively, assessed using the Kruskal-Wallis test and permutational multivariate analysis of variance (PERMANOVA) using 1000 permutations, performed through the *adonis* function implemented in *vegan* R package.^21^ Relative abundances were calculated at any taxonomic level (see **eTable 2**) and compared between *a priori* defined groups (see below) using the Kruskal-Wallis test. Furthermore, we computed Spearman’s rank correlation coefficient to measure the correlation between the concentration of C-ARDS laboratory variables (i.e., D-dimer, lactate dehydrogenase [LDH], or procalcitonin) and the relative abundance of lung microbiota-composing major phyla (i.e., Actinobacteria, Bacteroidetes, Firmicutes, or Proteobacteria). The strength and direction (negative or positive) of association between two ranked variables were measured by the ρ coefficient. Prior to Spearman’s correlation analysis, Gaussian distribution was assessed by the Shapiro–Wilk’s test.

Clinical data analysis was performed using R software 4.2.0 (R Core Team, 2022; Wien, Austria).^22^ Qualitative data were expressed as absolute and relative percentage frequency, whereas quantitative data as mean and standard deviation (SD), if Gaussian, or median and interquartile range (IQR), otherwise. Differences between *a priori* defined groups were assessed by either Student’s *t*-test or Mann-Whitney *U*-test (as appropriate) for quantitative data and by the chi-squared test or Fisher-Freeman-Halton’s exact test (as appropriate) for qualitative data. Gaussian distribution was previously evaluated by the Shapiro-Wilk’s test. Survival was measured starting from the date of hospital admission until all-cause death (event) or hospital discharge (censored), and Kaplan–Meier (KM) survival analysis was applied to assess potential differences in the composite outcome between *a priori* defined groups. Log-rank p and KM curves were further reported by means of “*ggplot2*” and “*survminer*” v0.4.9 R packages .^23,24^ Before investigation, Shannon diversity index was stratified into low (≤2.76) and high (>2.76) based on the composite outcome using “*maxstat*” R package.^25^ Uni- and multivariable Cox regression models were performed to assess for potential predictors of composite outcome, i.e., weaned and survived (as adjudicated at day 28 after BAL sampling or at hospital discharge). Ordinary proportional hazard Cox models were fitted by “*survival*” v3.5-5 R package,^26^ and hazard ratios (HRs) with 95% confidence intervals (CIs) were reported. Proportionality of hazard functions was assessed by the visual inspection of hazard plots and Schoenfeld residuals. When proportionality was doubtful, weighted Cox regression models were fitted.^27–30^ A multivariable Cox model was further fitted considering all variables emerged as significant/with known prognostic value at the ordinary Cox models. According to the rule to include one predictor at least every 10 events,^31^ otherwise suggestive findings were not considered due to the low number of events per variable in our study sample. Statistical significance was set at a p value <0.05. Suggestive findings were also reported (0.05 ≤ p < 0.10).

**Supplementary Results**

Baseline characteristics (including respiratory mechanics) upon BAL sampling and outcomes of C-ARDS patients (n=70) are summarized in **Table 1**, whereas additional characteristics (including VAP diagnosis) of the patients are presented in **eTable 1**. Thirty-five patients, of whom 10 had previously been studied,^9^ had a confirmed VAP diagnosis based on positive BAL cultures for *S. aureus* (n=15), *Acinetobacter baumannii* (n=11), or *Klebsiella pneumoniae* (n=9). Thirty-three (47.1%) patients had positive BAL cultures for *Candida* (n=23) or *Aspergillus* (n=10), while 38 (54.3%) and 34 (48.6%) patients were colonized by CRE or MRSA organisms, respectively. Fifty patients (71.4%) were treated with antimicrobial agents, mainly with macrolides (16/70, 22.9%) or beta-lactam (22/70, 31.4%) agents. After the first BAL sampling, six patients were subsequently sampled for suspected VAP, and BAL cultures yielded a bacterial etiology in all six cases.

Regarding two investigated parameters of respiratory mechanics, patients were categorized as having either a low (n=37) or high (n=33) Crs value, and a low (n=36) or high (n=34) VR value, respectively. Patients were further categorized based on the combination of Crs/VR parameters (see **eTable 2**), which led to define patient subgroups as follows: low-Crs/low-VR (n=24), low-Crs/high-VR (n=13), high-Crs/low-VR (n=12), and high-Crs/high-VR (n=21). However, the relatively small sample size of each subgroup prevented us from further specific investigations. We thus focused on the comparison of clinical, lung microbiota, and outcome variables in this study according to Crs and VR classifications, respectively (see **Table 1**).

Regarding lung microbiota diversity and composition analyses, we summarized the results of alfa diversity (see **eTable 3**), beta diversity (see **eTable 4**), and taxa relative abundance (see **Supplementary eTables 5 to 14**), as well as we provided graphical presentations of these results (see **Figures 1 and 2,** and **eFigures 3 to 7**).

Alfa-diversity measurements (see **eTable 3**) did not show any significant differences (Kruskal Wallis test) between low-Crs and high-Crs patients, as well as between low-VR and high-VR patients as for both observed species (p=0.075 for both comparisons) and Shannon diversity index (p=0.256 and p=0.805, respectively).

Beta-diversity measurements (see **eTable 4**) showed statistically significant differences (PERMANOVA test) at any taxonomic level for the low-Crs *vs* high-Crs patients’ comparison (phylum, p=0.012; class, p=0.015; order, p=0.028; family, p=0.004; and genus, p=0.008). Low-VR *vs* high-VR patients’ comparison instead disclosed statistically significant differences (PERMANOVA test) at levels of phylum (p=0.010), class (p=0.008), and order (p=0.034), whilst only suggestive at levels of family (p=0.077) and genus (p=0.072).

To explain these findings, rank abundance analyses of lung microbiota-composing taxa were performed for both low-Crs *vs* high-Crs patients’ (see **eTables 5 to 9**) and low-VR *vs* high-VR patients’ (see **eTables 10 to 14**) comparisons. The Kruskal-Wallis test showed that low-Crs patients’ samples significantly differed from high-Crs patients’ samples in terms of relative abundances at: (i) phylum level [Firmicutes (p=0.017) and Proteobacteria (p=0.043)] (see **eTable 5**); (ii) class level [Bacilli (p=0.030) and Gammaproteobacteria (p=0.025)] (see **eTable 6**),; (iii) order level [Bacillales (p=0.006)] (see **eTable 7**); (iv) family order [Lactobacillaceae (p=0.021), Moraxellaceae (p=0.041), and Paenibacillaceae (p=0.004)] (see **eTable 8**); or (v) genus level [*Acinetobacter* (p=0.042), *Lactobacillus* (p=0.024), and *Paenibacillus* (p=0.004)] (see **eTable 9**).

Likewise, low-VR patients’ samples significantly differed (Kruskal-Wallis test) from high-VR patients’ samples in terms of relative abundances at: (i) phylum level [Fusobacteria (p=0.015) and Firmicutes (p=0.014)] (see **eTable 10**); (ii) class level [Bacilli (p=0.021), Deltaproteobacteria (p=0.042), Fusobacteriia (p=0.015), and Gammaproteobacteria (p=0.004)] (see **eTable 11**); (iii) order level [Desulfovibrionales (p=0.042) and Fusobacteriales (p=0.015)] (see **eTable 12**); (iv) family order [Paenibacillaceae (p=0.037)] (see **Supplementary eTable 13**); or (v) genus level [*Paenibacillus* (p=0.037)] (see **eTable 14**).

Looking at the data from two between-group comparisons, we observed that taxa significantly differed between low-Crs and high-Crs patients’ samples at each of the five taxonomic levels, whilst suggestive findings were achieved between low-VR and high-VR patients’ samples [i.e., Firmicutes (p=0.073) at the phylum level, and Bacillales (p=0.054) at the order level]. Moreover, we further detected a suggestive difference between low-VR and high-VR patients’ samples at the order level (Pseudomonadales; p=0.057), family order (Pseudomonadaceae; p=0.070), and genus level (*Pseudomonas;* p=0.072).

Taken together, Proteobacteria (with the class Gammaproteobacteria) were relatively more abundant, and Firmicutes (with the class Bacilli) relatively less abundant in low-Crs (or low-VR) patients’ samples than in high-Crs (or high-VR) patients’ samples. Gammaproteobacteria (with the family Moraxellaceae and the genus *Acinetobacter*) were relatively more abundant in low-Crs patients’ samples than in high-Crs patients’ samples. Moreover, Bacilli (with the family Paenibacillaceae and the genus *Paenibacillus*) were relatively less abundant in low-Crs (or low-VR) patients’ samples than in high-Crs (or high-VR) patients’ samples. Of note, within Bacilli, the Lactobacillaceae family (with the genus *Lactobacillus*) was more abundant in low-Crs patients’ samples as compared to high-Crs patients’ samples.

The main outcome of the study was an in-hospital composite outcome, i.e. weaned from MV *and* survived, as assessed in terms of prognostic factors (see **Table 2**), and difference between low (≤2.76) or high (>2.76) Shannon diversity values stratified patients (see **Figure 5**). We further performed a Kaplan-Meier analysis to assess the cumulative incidence of the outcome after stratifying patients by VR or Crs/PBW values (see **eFigure 8**). As compared to patients with a VR >0.53 (n=34), patients with a VR ≤0.53 (n=36) had a lower, but not statistically significant, median time from BAL sampling to weaning from MV and survival (13 days and 19 days, respectively, log-rank p=0.700). Similarly, patients with CRS/PBW >2.2 (n=33) had a lower median time from BAL sampling to weaning from MV and survival than patients with a CRS/PBW ≤2.2 (n=37), though only a suggestive difference emerged (12 days and 25 days, respectively, log-rank p=0.057).

| **List of Supplementary Tables** | **Page** |
| --- | --- |
| **eTable 1.** Additional characteristics of C-ARDS patients at the time of BAL sampling (N=70). | 14 |
| **eTable 2.** Classification C-ARDS patients by respiratory system compliance (Crs), ventilatory ratio (VR), or combined Crs/VR parameters (N=70). | 15 |
| **eTable 3.** Comparison of lung microbiota alpha-diversity values as stratified according to respiratory system compliance and ventilatory ratio (N=70). | 16 |
| **eTable 4.** Lung microbiota taxonomic composition comparison based on the classification of the study cohort according to respiratory system compliance and ventilatory ratio (N=70). | 17 |
| **eTable 5.** Comparison of microbial phylum-level relative abundances in the study cohort, as stratified according to the compliance of the respiratory system (N=70). | 18 |
| **eTable 6.** Comparison of microbial class-level relative abundances in the study cohort, as stratified according to the compliance of the respiratory system (N=70). | 19 |
| **eTable 7.** Comparison of microbial order-level relative abundances in the study cohort, as stratified according to the compliance of the respiratory system (N=70). | 20 |
| **eTable 8.** Comparison of microbial family-level relative abundances in the study cohort, as stratified according to the compliance of the respiratory system (N=70). | 21 |
| **eTable 9.** Comparison of microbial genus-level relative abundances in the study cohort, as stratified according to the compliance of the respiratory system (N=70). | 22 |
| **eTable 10.** Comparison of microbial phylum-level relative abundances in the study cohort, as stratified according to the ventilatory ratio (N=70). | 23 |
| **eTable 11.** Comparison of microbial class-level relative abundances in the study cohort, as stratified according to the ventilatory ratio (N=70). | 24 |
| **eTable 12.** Comparison of microbial order-level relative abundances in the study cohort, as stratified according to the ventilatory ratio (N=70). | 25 |
| **eTable 13.** Comparison of microbial family-level relative abundances in the study cohort, as stratified according to the ventilatory ratio (N=70). | 26 |
| **eTable 14.** Comparison of microbial genus-level relative abundances in the study cohort, as stratified according to the ventilatory ratio (N=70). | 27 |

| **eTable 1. Additional characteristics of C-ARDS patients at the time of BAL sampling (N=70).** | |
| --- | --- |
|  | **No. (%)** |
| **Microbial species isolated from BAL cultures** |  |
| *Staphylococcus aureus* | 12 (17.1) |
| *Acinetobacter baumannii* | 10 (14.3) |
| *Klebsiella pneumoniae* | 8 (11.4) |
| *Pseudomonas aeruginosa* | 7 (10.0) |
| *Escherichia coli* | 4 (5.7) |
| Other bacterial species*** | 7 (10.0) |
| *Candida* species | 23 (32.9) |
| *Aspergillus* species | 10 (14.3) |
| **Microbiological diagnosis** |  |
| Ventilator-associated pneumonia*^#^* | 35 (50.0) |
| Urinary tract infection | 20 (28.6) |
| Bloodstream infection | 16 (22.9) |
| **Nasal/rectal colonization status due to:** |  |
| Carbapenem-resistant *Enterobacterales* | 38 (54.3) |
| Methicillin-resistant *S. aureus* | 34 (48.6) |
| **Ongoing antimicrobial treatment(s)** | 50 (71.4) |
| Macrolides | 16 (22.9) |
| Cephalosporins | 12 (17.1) |
| Beta-lactams *plus* beta-lactamase inhibitors | 10 (14.3) |
| Tigecycline | 5 (7.1) |
| Linezolid | 5 (7.1) |
| Fluoroquinolones | 2 (2.9) |
| **Abbreviations.** C-ARDS: COVID-19-related ARDS; BAL: bronchoalveolar lavage.  ***Include *Stenotrophomonas maltophilia* (n=2), *Citrobacter freundii* (n=1), *Klebsiella aerogenes* (n=1), *Klebsiella oxytoca* (n=1), *Proteus mirabilis* (n=1), and *Streptococcus pneumoniae* (n=1).  *^#^*Thirteen (37.1%) out of 35 episodes had a polymicrobial etiology due to the combination of following organisms: *Acinetobacter baumannii* (n=8), *Staphylococcus aureus* (n=4), *Klebsiella pneumoniae* (n=4), *Escherichia coli* (n=3), *Pseudomonas aeruginosa* (n=2), *Stenotrophomonas maltophilia* (n=2), *Klebsiella oxytoca* (n=1), *Citrobacter freundii* (n=1), or *Proteus mirabilis* (n=1). | |

| **eTable 2.** Classification of C-ARDS patients by respiratory system compliance (Crs), ventilatory ratio (VR) and combined Crs/VR parameters (N=70).***^*^*** | |
| --- | --- |
| **Category** | **No. of patients** |
| **Single parameter** |  |
| Crs, low value | 37 |
| Crs, high value | 33 |
| VR, low value | 36 |
| VR, high value | 34 |
| **Double parameter** |  |
| Low-Crs/Low-VR | 24 |
| Low-Crs/High-VR | 13 |
| High-Crs/Low-VR | 12 |
| High-Crs/High-VR | 21 |
| **Abbreviations.** Crs: respiratory system compliance. VR: Ventilatory ratio.  ** Patients were categorized as “low-Crs” or “high-Crs” according to their mean respiratory system compliance/predicted body weight (Crs/PBW) values, respectively ≤0.53 (mL/cmH_2_O)/kg (low) and >0.53 (mL/cmH_2_O)/kg (high). Patients were instead classified as “low-VR” or “high-VR” according to their median value, respectively ≤2.2 (low) or >2.2 (high). Patients were further categorized according to the combinations of Crs (low or high)/VR (low or high), as indicated. | |

| **eTable 3.** Comparison of lung microbiota alpha-diversity values as stratified according to respiratory system compliance and ventilatory ratio (N=70). | | | | | | |
| --- | --- | --- | --- | --- | --- | --- |
|  | **Low-Crs** | **High-Crs** | **p** | **Low-VR** | **High-VR** | **p** |
|  | **N=37** | **N=33** |  | **N=36** | **N=34** |  |
| **Observed species** |  |  |  |  |  |  |
| Mean (±SD) | 69.6 (38.7) | 54.1 (42.9) | 0.075 | 67.5 (39.2) | 56.8 (43.0) | 0.075 |
| Median (IQR) | 60 (36–96) | 71 (15–86) |  | 58.8 (35.7–94.5) | 75.3 (15.7–91.0) |  |
| **Shannon diversity** |  |  |  |  |  |  |
| Mean (±SD) | 1.8 (0.9) | 2.0 (0.8) | 0.256 | 1.9 (0.9) | 1.9 (0.8) | 0.805 |
| Median (IQR) | 1.3 (1.2–2.5) | 1.3 (1.3–2.6) |  | 1.4 (1.2–2.6) | 1.2 (1.3–2.5) |  |
| **Abbreviations**. Crs: =compliance of the respiratory system; VR: ventilatory ratio; SD: standard deviation; IQR: interquartile range. | | | | | | |

| **eTable 4.** Lung microbiota taxonomic composition comparison based on the classification of the study cohort according to respiratory system compliance and ventilatory ratio (N=70). | | |
| --- | --- | --- |
|  | **PERMANOVA (p-values)** | |
| **Taxonomic level** | **low-Crs (n=37) *vs* high-Crs (n=33)** | **low-VR (n=36) *vs* high-VR (n=34)** |
| Phylum | **0.012** | **0.010** |
| Class | **0.015** | **0.008** |
| Order | **0.028** | **0.034** |
| Family | **0.004** | *0.077* |
| Genus | **0.008** | *0.072* |
| ASV | **0.008** | *0.082* |
| **Abbreviations.** PERMANOVA: permutational multivariate analysis of variance; Crs: compliance of the respiratory system; VR: ventilatory ratio; ASV: amplicon sequence variant.  * Calculated using the *vegan* package’s *adonis* function,^21^ requiring 1000 permutations. In bold significant findings (p<0.05), in italic the suggestive ones (0.05 ≤ p <0.10). | | |

| **eTable 5.** Comparison of microbial phylum-level relative abundances in the study cohort, as stratified according to the compliance of the respiratory system (N=70). | | | | | | | |
| --- | --- | --- | --- | --- | --- | --- | --- |
| **Phylum** | **Low-Crs (N=37)** | | | **High-Crs (N=33)** | | | **p*** |
|  | **Mean** | **SD** | **SE** | **Mean** | **SD** | **SE** |  |
| Actinobacteria | 0.078 | 0.145 | 0.024 | 0.041 | 0.057 | 0.010 | 0.655 |
| Bacteroidetes | 0.081 | 0.097 | 0.016 | 0.120 | 0.108 | 0.019 | 0.126 |
| Firmicutes | 0.477 | 0.334 | 0.055 | 0.661 | 0.251 | 0.044 | **0.017** |
| Fusobacteria | 0.002 | 0.003 | 0.001 | 0.007 | 0.020 | 0.003 | 0.674 |
| Proteobacteria | 0.362 | 0.382 | 0.063 | 0.172 | 0.270 | 0.047 | **0.043** |
| **Abbreviations.** Crs: compliance of the respiratory system; SD: standard deviation; SE: standard error.  * P-values were computed by the Kruskal-Wallis test (in bold significant findings, i.e. p<0.05). | | | | | | | |

| **eTable 6.** Comparison of microbial class-level relative abundances in the study cohort, as stratified according to the compliance of the respiratory system (N=70). | | | | | | | |
| --- | --- | --- | --- | --- | --- | --- | --- |
| **Class** | **Low-Crs (N=37)** | | | **High-Crs (N=33)** | | | **p*** |
|  | **Mean** | **SD** | **SE** | **Mean** | **SD** | **SE** |  |
| Actinobacteria | 0.074 | 0.145 | 0.024 | 0.038 | 0.056 | 0.010 | 0.746 |
| Alphaproteobacteria | 0.004 | 0.015 | 0.002 | 0.011 | 0.039 | 0.007 | 0.597 |
| Bacilli | 0.380 | 0.282 | 0.046 | 0.519 | 0.289 | 0.050 | **0.030** |
| Bacteroidia | 0.081 | 0.097 | 0.016 | 0.120 | 0.108 | 0.019 | 0.125 |
| Clostridia | 0.084 | 0.118 | 0.019 | 0.126 | 0.139 | 0.024 | 0.188 |
| Coriobacteriia | 0.004 | 0.006 | 0.001 | 0.003 | 0.007 | 0.001 | 0.277 |
| Deltaproteobacteria | 0.000 | 0.001 | 0.000 | 0.000 | 0.002 | 0.000 | 0.257 |
| Erysipelotrichia | 0.002 | 0.004 | 0.001 | 0.006 | 0.017 | 0.003 | 0.800 |
| Fusobacteriia | 0.002 | 0.003 | 0.001 | 0.007 | 0.020 | 0.003 | 0.669 |
| Gammaproteobacteria | 0.358 | 0.383 | 0.063 | 0.161 | 0.272 | 0.047 | **0.025** |
| Negativicutes | 0.011 | 0.032 | 0.005 | 0.010 | 0.016 | 0.003 | 0.943 |
| **Abbreviations.** Crs: compliance of the respiratory system; SD: standard deviation; SE: standard error.  * P-values were computed by the Kruskal-Wallis test (in bold significant findings, i.e. p<0.05). | | | | | | | |

| **eTable 7.** Comparison of microbial order-level relative abundances in the study cohort, as stratified according to the compliance of the respiratory system (N=70). | | | | | | | |
| --- | --- | --- | --- | --- | --- | --- | --- |
| **Order** | **Low-Crs (N=37)** | | | **High-Crs (N=33)** | | | **p*** |
|  | **Mean** | **SD** | **SE** | **Mean** | **SD** | **SE** |  |
| Actinomycetales | 0.000 | 0.000 | 0.000 | 0.000 | 0.001 | 0.000 | 0.451 |
| Bacillales | 0.133 | 0.239 | 0.039 | 0.277 | 0.316 | 0.055 | **0.006** |
| Bacteroidales | 0.081 | 0.097 | 0.016 | 0.120 | 0.108 | 0.019 | 0.125 |
| Betaproteobacteriales | 0.003 | 0.006 | 0.001 | 0.005 | 0.013 | 0.002 | 0.914 |
| Bifidobacteriales | 0.013 | 0.019 | 0.003 | 0.013 | 0.021 | 0.004 | 0.331 |
| Class_Bacteroidia | 0.000 | 0.000 | 0.000 | 0.000 | 0.000 | 0.000 | 0.930 |
| Clostridiales | 0.084 | 0.118 | 0.012 | 0.126 | 0.139 | 0.024 | 0.188 |
| Coriobacteriales | 0.004 | 0.006 | 0.001 | 0.003 | 0.007 | 0.001 | 0.277 |
| Corynebacteriales | 0.057 | 0.149 | 0.024 | 0.011 | 0.035 | 0.006 | 0.124 |
| Desulfovibrionales | 0.001 | 0.001 | 0.000 | 0.001 | 0.002 | 0.000 | 0.257 |
| Enterobacteriales | 0.076 | 0.192 | 0.032 | 0.039 | 0.092 | 0.016 | 0.461 |
| Erysipelotrichales | 0.002 | 0.004 | 0.001 | 0.006 | 0.017 | 0.003 | 0.800 |
| Fusobacteriales | 0.002 | 0.003 | 0.001 | 0.007 | 0.020 | 0.004 | 0.669 |
| Lactobacillales | 0.247 | 0.259 | 0.043 | 0.242 | 0.256 | 0.045 | 0.864 |
| Micrococcales | 0.002 | 0.007 | 0.001 | 0.000 | 0.001 | 0.000 | *0.087* |
| Pasteurellales | 0.012 | 0.069 | 0.011 | 0.005 | 0.031 | 0.005 | 0.625 |
| Propionibacteriales | 0.002 | 0.006 | 0.001 | 0.013 | 0.031 | 0.005 | *0.097* |
| Pseudomonadales | 0.268 | 0.383 | 0.063 | 0.111 | 0.266 | 0.046 | *0.093* |
| Rhizobiales | 0.000 | 0.001 | 0.000 | 0.001 | 0.005 | 0.001 | 0.136 |
| Rhodospirillales | 0.000 | 0.001 | 0.000 | 0.004 | 0.015 | 0.003 | 0.534 |
| Selenomonadales | 0.011 | 0.032 | 0.005 | 0.010 | 0.016 | 0.003 | 0.943 |
| Sphingomonadales | 0.003 | 0.014 | 0.002 | 0.006 | 0.024 | 0.004 | 0.856 |
| **Abbreviations.** Crs: compliance of the respiratory system; SD: standard deviation; SE: standard error.  * P-values were computed by the Kruskal-Wallis test. In bold significant findings (p<0.05), in italic the suggestive ones (0.05 ≤ p <0.10). | | | | | | | |

| **eTable 8.** Comparison of microbial family-level relative abundances in the study cohort, as stratified according to the compliance of the respiratory system (N=70). | | | | | | | |
| --- | --- | --- | --- | --- | --- | --- | --- |
| **Family** | **Low-Crs (N=37)** | | | **High-Crs (N=33)** | | | **p*** |
|  | **Mean** | **SD** | **SE** | **Mean** | **SD** | **SE** |  |
| Bacteroidaceae | 0.023 | 0.038 | 0.006 | 0.064 | 0.091 | 0.016 | 0.292 |
| Bifidobacteriaceae | 0.014 | 0.021 | 0.003 | 0.015 | 0.025 | 0.004 | 0.337 |
| Carnobacteriaceae | 0.010 | 0.034 | 0.006 | 0.018 | 0.055 | 0.010 | 0.461 |
| Corynebacteriaceae | 0.057 | 0.150 | 0.025 | 0.011 | 0.035 | 0.006 | 0.131 |
| Enterobacteriaceae | 0.077 | 0.193 | 0.032 | 0.041 | 0.095 | 0.017 | 0.490 |
| Enterococcaceae | 0.003 | 0.008 | 0.001 | 0.008 | 0.021 | 0.004 | 0.569 |
| Family XI | 0.026 | 0.108 | 0.018 | 0.045 | 0.114 | 0.020 | 0.771 |
| Lachnospiraceae | 0.030 | 0.041 | 0.007 | 0.037 | 0.051 | 0.009 | 0.754 |
| Lactobacillaceae | 0.194 | 0.230 | 0.038 | 0.115 | 0.202 | 0.035 | **0.021** |
| Moraxellaceae | 0.226 | 0.370 | 0.061 | 0.043 | 0.168 | 0.029 | **0.041** |
| Paenibacillaceae | 0.049 | 0.160 | 0.026 | 0.182 | 0.278 | 0.048 | **0.004** |
| Pasteurellaceae | 0.013 | 0.070 | 0.011 | 0.007 | 0.037 | 0.007 | 0.625 |
| Peptostreptococcaceae | 0.009 | 0.023 | 0.004 | 0.020 | 0.045 | 0.008 | 0.919 |
| Prevotellaceae | 0.046 | 0.088 | 0.014 | 0.052 | 0.077 | 0.013 | 0.846 |
| Propionibacteriaceae | 0.003 | 0.006 | 0.001 | 0.014 | 0.036 | 0.006 | 0.100 |
| Pseudomonadaceae | 0.045 | 0.170 | 0.028 | 0.069 | 0.224 | 0.039 | 0.579 |
| Ruminococcaceae | 0.024 | 0.031 | 0.005 | 0.040 | 0.061 | 0.011 | 0.827 |
| Sphingomonadaceae | 0.003 | 0.014 | 0.002 | 0.007 | 0.026 | 0.005 | 0.856 |
| Staphylococcaceae | 0.082 | 0.194 | 0.032 | 0.095 | 0.211 | 0.037 | 0.245 |
| Streptococcaceae | 0.058 | 0.139 | 0.023 | 0.116 | 0.189 | 0.033 | 0.411 |
| **Abbreviations.** Crs: compliance of the respiratory system; SD: standard deviation; SE: standard error.  * P-values were computed by the Kruskal-Wallis test (in bold significant findings, i.e. p<0.05). | | | | | | | |

| **eTable 9.** Comparison of microbial genus-level relative abundances in the study cohort, as stratified according to the compliance of the respiratory system (N=70). | | | | | | | |
| --- | --- | --- | --- | --- | --- | --- | --- |
| **Genus** | **Low-Crs (N=37)** | | | **High-Crs (N=33)** | | | **p*** |
|  | **Mean** | **SD** | **SE** | **Mean** | **SD** | **SE** |  |
| Acinetobacter | 0.232 | 0.377 | 0.062 | 0.047 | 0.178 | 0.031 | **0.042** |
| Bacteroides | 0.035 | 0.046 | 0.008 | 0.076 | 0.112 | 0.020 | 0.303 |
| Bifidobacterium | 0.017 | 0.026 | 0.004 | 0.017 | 0.033 | 0.006 | 0.198 |
| Corynebacterium 1 | 0.059 | 0.153 | 0.025 | 0.012 | 0.037 | 0.007 | 0.128 |
| Cutibacterium | 0.003 | 0.006 | 0.001 | 0.018 | 0.049 | 0.009 | 0.107 |
| Escherichia-Shigella | 0.023 | 0.083 | 0.014 | 0.023 | 0.084 | 0.015 | 0.519 |
| Faecalibacterium | 0.014 | 0.023 | 0.004 | 0.029 | 0.057 | 0.010 | 0.718 |
| Finegoldia | 0.019 | 0.111 | 0.018 | 0.018 | 0.057 | 0.010 | 0.587 |
| Granulicatella | 0.011 | 0.035 | 0.006 | 0.018 | 0.056 | 0.010 | 0.453 |
| Haemophilus | 0.013 | 0.072 | 0.012 | 0.008 | 0.047 | 0.008 | 0.613 |
| Klebsiella | 0.056 | 0.185 | 0.030 | 0.016 | 0.061 | 0.011 | 0.141 |
| Lactobacillus | 0.227 | 0.275 | 0.045 | 0.135 | 0.239 | 0.042 | **0.024** |
| Novosphingobium | 0.002 | 0.009 | 0.002 | 0.006 | 0.026 | 0.005 | 0.856 |
| Paenibacillus | 0.051 | 0.164 | 0.027 | 0.192 | 0.285 | 0.050 | **0.004** |
| Peptostreptococcus | 0.009 | 0.024 | 0.004 | 0.023 | 0.067 | 0.012 | 0.886 |
| Prevotella | 0.009 | 0.041 | 0.007 | 0.017 | 0.057 | 0.010 | 0.894 |
| Prevotella 7 | 0.025 | 0.082 | 0.014 | 0.034 | 0.069 | 0.012 | 0.391 |
| Pseudomonas | 0.047 | 0.175 | 0.029 | 0.072 | 0.231 | 0.040 | 0.590 |
| Staphylococcus | 0.086 | 0.201 | 0.033 | 0.106 | 0.233 | 0.041 | 0.226 |
| Streptococcus | 0.064 | 0.151 | 0.025 | 0.132 | 0.215 | 0.037 | 0.323 |
| **Abbreviations.** Crs: compliance of the respiratory system; SD: standard deviation; SE: standard error.  * P-values were computed by the Kruskal-Wallis test (in bold significant findings, i.e. p<0.05). | | | | | | | |

| **eTable 10.** Comparison of microbial phylum-level relative abundances in the study cohort, as stratified according to the ventilatory ratio (N=70). | | | | | | | |
| --- | --- | --- | --- | --- | --- | --- | --- |
| **Phylum** | **Low-VR (N=36)** | | | **High-VR (N=34)** | | | **p*** |
|  | **Mean** | **SD** | **SE** | **Mean** | **SD** | **SE** |  |
| Actinobacteria | 0.045 | 0.080 | 0.013 | 0.078 | 0.140 | 0.024 | 0.247 |
| Bacteroidetes | 0.086 | 0.084 | 0.014 | 0.114 | 0.120 | 0.021 | 0.573 |
| Firmicutes | 0.485 | 0.342 | 0.057 | 0.647 | 0.250 | 0.043 | *0.073* |
| Fusobacteria | 0.005 | 0.014 | 0.002 | 0.003 | 0.014 | 0.003 | **0.015** |
| Proteobacteria | 0.380 | 0.381 | 0.063 | 0.159 | 0.263 | 0.045 | **0.014** |
| **Abbreviations.** VR: ventilatory ratio; SD: standard deviation; SE: standard error.  * P-values were computed by the Kruskal-Wallis test. In bold significant findings (p<0.05), in italic the suggestive ones (0.05 ≤ p <0.10). | | | | | | | |

| **eTable 11.** Comparison of microbial class-level relative abundances in the study cohort, as stratified according to the ventilatory ratio (N=70). | | | | | | | |
| --- | --- | --- | --- | --- | --- | --- | --- |
| **Class** | **Low-VR (N=36)** | | | **High-VR (N=34)** | | | **p*** |
|  | **Mean** | **SD** | **SE** | **Mean** | **SD** | **SE** |  |
| Actinobacteria | 0.042 | 0.079 | 0.013 | 0.073 | 0.140 | 0.024 | 0.301 |
| Alphaproteobacteria | 0.003 | 0.014 | 0.002 | 0.011 | 0.038 | 0.007 | 0.813 |
| Bacilli | 0.365 | 0.301 | 0.050 | 0.531 | 0.259 | 0.044 | **0.021** |
| Bacteroidia | 0.086 | 0.084 | 0.014 | 0.114 | 0.120 | 0.021 | 0.569 |
| Clostridia | 0.102 | 0.152 | 0.025 | 0.105 | 0.102 | 0.017 | 0.335 |
| Coriobacteriia | 0.003 | 0.004 | 0.001 | 0.004 | 0.008 | 0.001 | 0.995 |
| Deltaproteobacteria | 0.001 | 0.002 | 0.000 | 0.000 | 0.001 | 0.000 | **0.042** |
| Erysipelotrichia | 0.003 | 0.006 | 0.001 | 0.005 | 0.016 | 0.003 | 0.154 |
| Fusobacteriia | 0.005 | 0.014 | 0.002 | 0.003 | 0.014 | 0.002 | **0.015** |
| Gammaproteobacteria | 0.376 | 0.381 | 0.064 | 0.148 | 0.265 | 0.045 | **0.004** |
| Negativicutes | 0.015 | 0.034 | 0.006 | 0.006 | 0.007 | 0.001 | 0.162 |
| **Abbreviations.** VR: ventilatory ratio; SD: standard deviation; SE: standard error.  * P-values were computed by the Kruskal-Wallis test (in bold significant findings, i.e. p<0.05). | | | | | | | |

| **eTable 12.** Comparison of microbial order-level relative abundances in the study cohort, as stratified according to the ventilatory ratio (N=70). | | | | | | | |
| --- | --- | --- | --- | --- | --- | --- | --- |
| **Order** | **Low-VR (N=36)** | | | **High-VR (N=34)** | | | **p*** |
|  | **Mean** | **SD** | **SE** | **Mean** | **SD** | **SE** |  |
| Actinomycetales | 0.000 | 0.001 | 0.000 | 0.000 | 0.001 | 0.000 | 0.510 |
| Bacillales | 0.130 | 0.241 | 0.040 | 0.275 | 0.312 | 0.054 | *0.054* |
| Bacteroidales | 0.086 | 0.084 | 0.014 | 0.114 | 0.120 | 0.021 | 0.569 |
| Betaproteobacteriales | 0.005 | 0.013 | 0.002 | 0.003 | 0.006 | 0.001 | 0.131 |
| Bifidobacteriales | 0.016 | 0.023 | 0.004 | 0.010 | 0.017 | 0.003 | 0.154 |
| Class_Bacteroidia | 0.000 | 0.000 | 0.000 | 0.000 | 0.000 | 0.000 | 0.977 |
| Clostridiales | 0.102 | 0.152 | 0.025 | 0.105 | 0.102 | 0.018 | 0.335 |
| Coriobacteriales | 0.003 | 0.004 | 0.001 | 0.004 | 0.008 | 0.001 | 0.995 |
| Corynebacteriales | 0.016 | 0.068 | 0.011 | 0.055 | 0.144 | 0.025 | 0.658 |
| Desulfovibrionales | 0.001 | 0.002 | 0.000 | 0.000 | 0.001 | 0.000 | **0.042** |
| Enterobacteriales | 0.073 | 0.192 | 0.032 | 0.043 | 0.096 | 0.016 | 0.777 |
| Erysipelotrichales | 0.003 | 0.006 | 0.001 | 0.005 | 0.016 | 0.003 | 0.154 |
| Fusobacteriales | 0.005 | 0.014 | 0.002 | 0.003 | 0.014 | 0.003 | **0.015** |
| Lactobacillales | 0.234 | 0.259 | 0.043 | 0.256 | 0.257 | 0.044 | 0.787 |
| Micrococcales | 0.001 | 0.002 | 0.000 | 0.002 | 0.007 | 0.001 | 0.172 |
| Pasteurellales | 0.018 | 0.075 | 0.012 | 0.000 | 0.001 | 0.000 | 0.847 |
| Propionibacteriales | 0.009 | 0.029 | 0.005 | 0.006 | 0.012 | 0.002 | 0.812 |
| Pseudomonadales | 0.280 | 0.387 | 0.064 | 0.102 | 0.258 | 0.044 | *0.057* |
| Rhizobiales | 0.001 | 0.005 | 0.001 | 0.000 | 0.000 | 0.000 | *0.097* |
| Rhodospirillales | 0.000 | 0.001 | 0.000 | 0.004 | 0.014 | 0.003 | 0.591 |
| Selenomonadales | 0.015 | 0.034 | 0.006 | 0.006 | 0.007 | 0.001 | 0.162 |
| Sphingomonadales | 0.002 | 0.012 | 0.002 | 0.007 | 0.025 | 0.004 | *0.084* |
| **Abbreviations.** VR: ventilatory ratio; SD: standard deviation; SE: standard error.  * P-values were computed by the Kruskal-Wallis test. In bold significant findings (p<0.05), in italic the suggestive ones (0.05 ≤ p < 0.10)B. | | | | | | | |

| **eTable 13.** Comparison of microbial family-level relative abundances in the study cohort, as stratified according to the ventilatory ratio (N=70). | | | | | | | |
| --- | --- | --- | --- | --- | --- | --- | --- |
| **Family** | **Low-VR (n=36)** | | | **High-VR (n=34)** | | | **p*** |
|  | **Mean** | **SD** | **SE** | **Mean** | **SD** | **SE** |  |
| Bacteroidaceae | 0.042 | 0.057 | 0.010 | 0.049 | 0.082 | 0.014 | 0.972 |
| Bifidobacteriaceae | 0.017 | 0.026 | 0.004 | 0.011 | 0.019 | 0.003 | 0.157 |
| Carnobacteriaceae | 0.011 | 0.033 | 0.005 | 0.017 | 0.055 | 0.009 | 0.687 |
| Corynebacteriaceae | 0.017 | 0.068 | 0.011 | 0.056 | 0.145 | 0.025 | 0.668 |
| Enterobacteriaceae | 0.075 | 0.194 | 0.032 | 0.045 | 0.099 | 0.017 | 0.804 |
| Enterococcaceae | 0.004 | 0.009 | 0.001 | 0.007 | 0.021 | 0.004 | 0.767 |
| Family XI | 0.041 | 0.131 | 0.022 | 0.029 | 0.085 | 0.015 | 0.914 |
| Lachnospiraceae | 0.037 | 0.053 | 0.009 | 0.030 | 0.038 | 0.007 | 0.841 |
| Lactobacillaceae | 0.171 | 0.225 | 0.037 | 0.142 | 0.216 | 0.037 | 0.181 |
| Moraxellaceae | 0.211 | 0.355 | 0.059 | 0.065 | 0.221 | 0.038 | 0.122 |
| Paenibacillaceae | 0.066 | 0.188 | 0.031 | 0.160 | 0.265 | 0.046 | **0.037** |
| Pasteurellaceae | 0.019 | 0.078 | 0.013 | 0.000 | 0.001 | 0.000 | 0.847 |
| Peptostreptococcaceae | 0.013 | 0.038 | 0.006 | 0.016 | 0.033 | 0.006 | 0.720 |
| Prevotellaceae | 0.037 | 0.061 | 0.010 | 0.061 | 0.099 | 0.017 | 0.851 |
| Propionibacteriaceae | 0.010 | 0.034 | 0.006 | 0.006 | 0.012 | 0.002 | 0.822 |
| Pseudomonadaceae | 0.074 | 0.230 | 0.038 | 0.038 | 0.153 | 0.026 | *0.070* |
| Ruminococcaceae | 0.027 | 0.040 | 0.007 | 0.037 | 0.055 | 0.010 | 0.711 |
| Sphingomonadaceae | 0.002 | 0.012 | 0.002 | 0.008 | 0.026 | 0.005 | *0.084* |
| Staphylococcaceae | 0.059 | 0.171 | 0.029 | 0.119 | 0.227 | 0.039 | 0.471 |
| Streptococcaceae | 0.069 | 0.154 | 0.026 | 0.104 | 0.178 | 0.031 | 0.504 |
| **Abbreviations.** VR: ventilatory ratio; SD: standard deviation; SE: standard error.  * P-values were computed by the Kruskal-Wallis test. In bold significant findings (p<0.05), in italic the suggestive ones (0.05 ≤ p < 0.10). | | | | | | | |

| **eTable 14.** Comparison of microbial genus-level relative abundances in the study cohort, as stratified according to the ventilatory ratio (N=70). | | | | | | | |
| --- | --- | --- | --- | --- | --- | --- | --- |
| **Genus** | **Low-VR (N=36)** | | | **High-VR (N=34)** | | | **p*** |
|  | **Mean** | **SD** | **SE** | **Mean** | **SD** | **SE** |  |
| Acinetobacter | 0.219 | 0.365 | 0.061 | 0.066 | 0.223 | 0.038 | 0.116 |
| Bacteroides | 0.052 | 0.077 | 0.013 | 0.057 | 0.095 | 0.016 | 0.944 |
| Bifidobacterium | 0.022 | 0.034 | 0.006 | 0.012 | 0.022 | 0.004 | 0.083 |
| Corynebacterium 1 | 0.017 | 0.069 | 0.012 | 0.057 | 0.149 | 0.026 | 0.688 |
| Cutibacterium | 0.014 | 0.047 | 0.008 | 0.007 | 0.013 | 0.002 | 0.822 |
| Escherichia-Shigella | 0.025 | 0.084 | 0.014 | 0.021 | 0.082 | 0.014 | 0.471 |
| Faecalibacterium | 0.016 | 0.032 | 0.005 | 0.026 | 0.052 | 0.009 | 0.897 |
| Finegoldia | 0.021 | 0.113 | 0.019 | 0.016 | 0.055 | 0.010 | 0.400 |
| Granulicatella | 0.011 | 0.034 | 0.006 | 0.018 | 0.056 | 0.010 | 0.687 |
| Haemophilus | 0.021 | 0.085 | 0.014 | 0.000 | 0.001 | 0.000 | 0.847 |
| Klebsiella | 0.048 | 0.185 | 0.031 | 0.025 | 0.073 | 0.013 | 1.000 |
| Lactobacillus | 0.201 | 0.267 | 0.045 | 0.164 | 0.257 | 0.044 | 0.170 |
| Novosphingobium | 0.001 | 0.008 | 0.001 | 0.007 | 0.026 | 0.004 | *0.084* |
| Paenibacillus | 0.069 | 0.190 | 0.032 | 0.168 | 0.274 | 0.047 | **0.037** |
| Peptostreptococcus | 0.016 | 0.060 | 0.010 | 0.016 | 0.035 | 0.006 | 0.920 |
| Prevotella | 0.014 | 0.046 | 0.008 | 0.012 | 0.053 | 0.009 | 0.492 |
| Prevotella 7 | 0.013 | 0.038 | 0.006 | 0.047 | 0.099 | 0.017 | 0.426 |
| Pseudomonas | 0.078 | 0.239 | 0.040 | 0.038 | 0.156 | 0.027 | *0.072* |
| Staphylococcus | 0.063 | 0.176 | 0.029 | 0.130 | 0.249 | 0.043 | 0.485 |
| Streptococcus | 0.079 | 0.174 | 0.029 | 0.114 | 0.198 | 0.034 | 0.599 |
| **Abbreviations.** VR: ventilatory ratio; SD: standard deviation; SE: standard error.  * P-values were computed by the Kruskal-Wallis test. In bold significant findings (p<0.05), in italic the suggestive ones (0.05 ≤ p < 0.10). | | | | | | | |


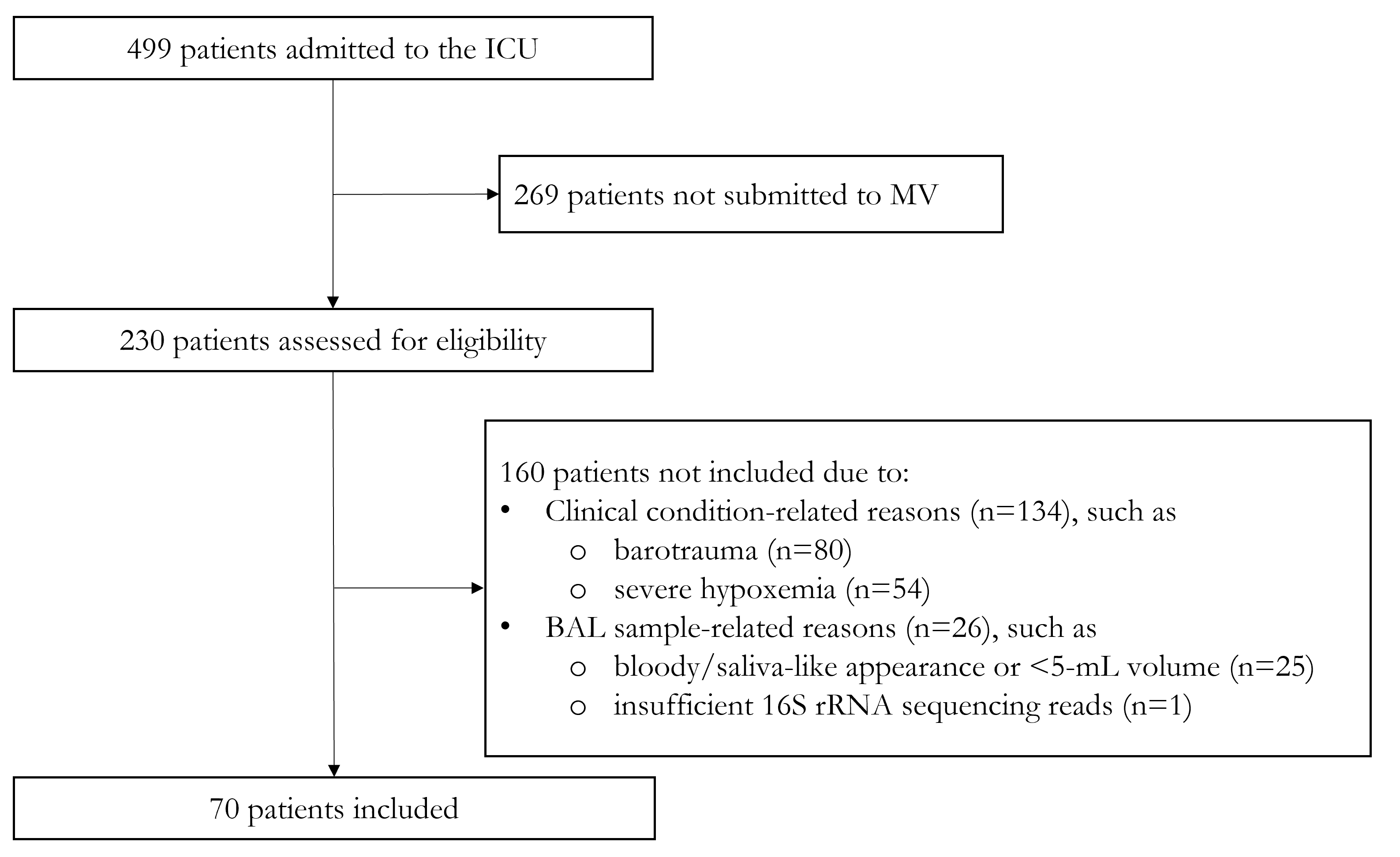


**eFigure 1. Flow chart of the study, with relative BAL samples.** Patients from whom the first BAL fluid sample since ICU admission was available for lung microbiota analysis were enrolled in the study (n=70), according to the selection process specified in the flowchart. Out of 230 eligible patients, 160 could not be included due to either clinical (n=134) or sample-related (n=26) reasons. One patient was excluded as his/her BAL fluid sample contained a low sequence amount (n=1000). [**Abbreviations**. ICU: Intensive Care Unit; MV: mechanical ventilation].

**
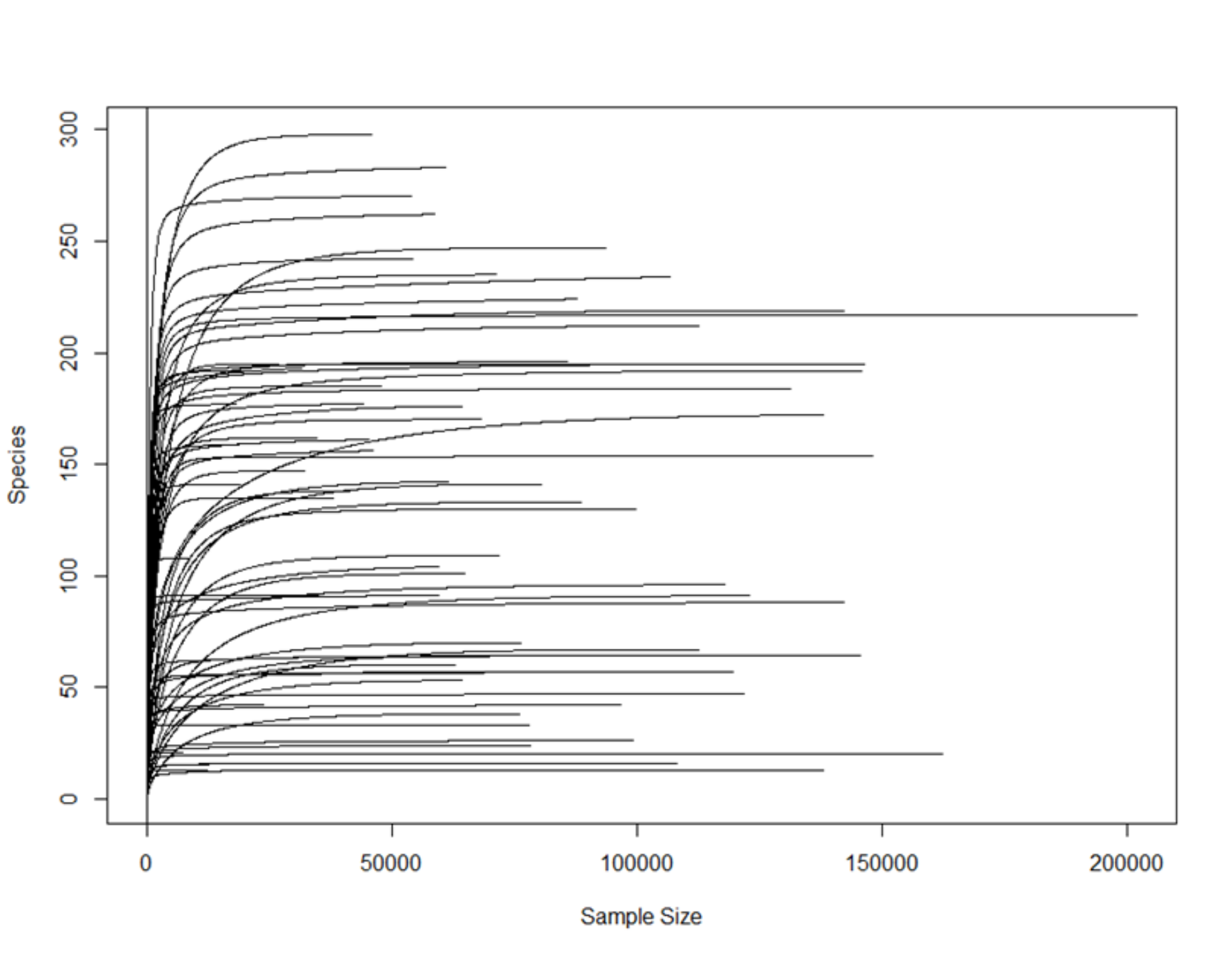
**

**eFigure 2. Sequencing depth determination for BAL fluid samples prior to lung microbiota downstream analyses.** Rarefaction curve shows the number of sequences generated through 16S rRNA gene amplification *versus* the number of species identified in each BAL fluid sample at a cutoff of 99% sequence similarity. As depicted, a sufficient depth of sequencing was achieved for each sample. Overall (N=70), after excluding one sample with a too low number of sequences, 3.908.676 sequences (corresponding to 455 taxa) were available for downstream analyses of lung microbiota (i.e., either alfa-diversity or beta-diversity).

**
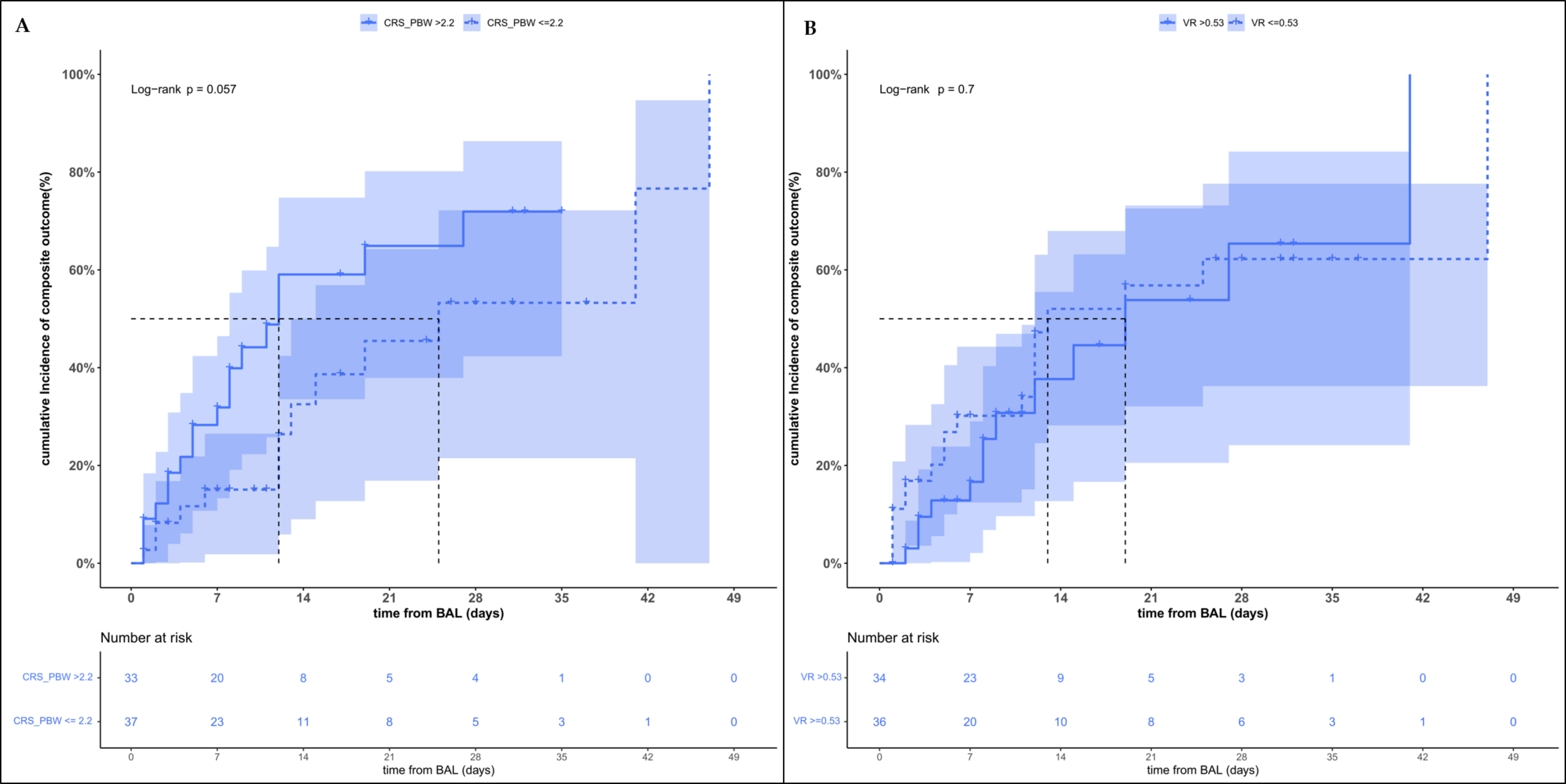
**

**eFigure 3. Cumulative incidence of in-hospital composite outcome (weaned and survived) for patients stratified according to low- or high-VR values, and low- or high-Crs values, respectively.** Kaplan-Meier curve analysis showed none significant difference between low- and high-VR patients (log-rank p=0.700) (**Panel A**) in terms of median time from BAL sampling to weaning from MV and survival at hospital discharge. Indeed, low-Crs disclosed a suggestive difference as compared to high-Crs subgroup (log-rank p=0.057) (**Panel B**). [**Abbreviations**. Crs: compliance of respiratory system; VR: Ventilatory Ratio; BAL: bronchoalveolar lavage].

**
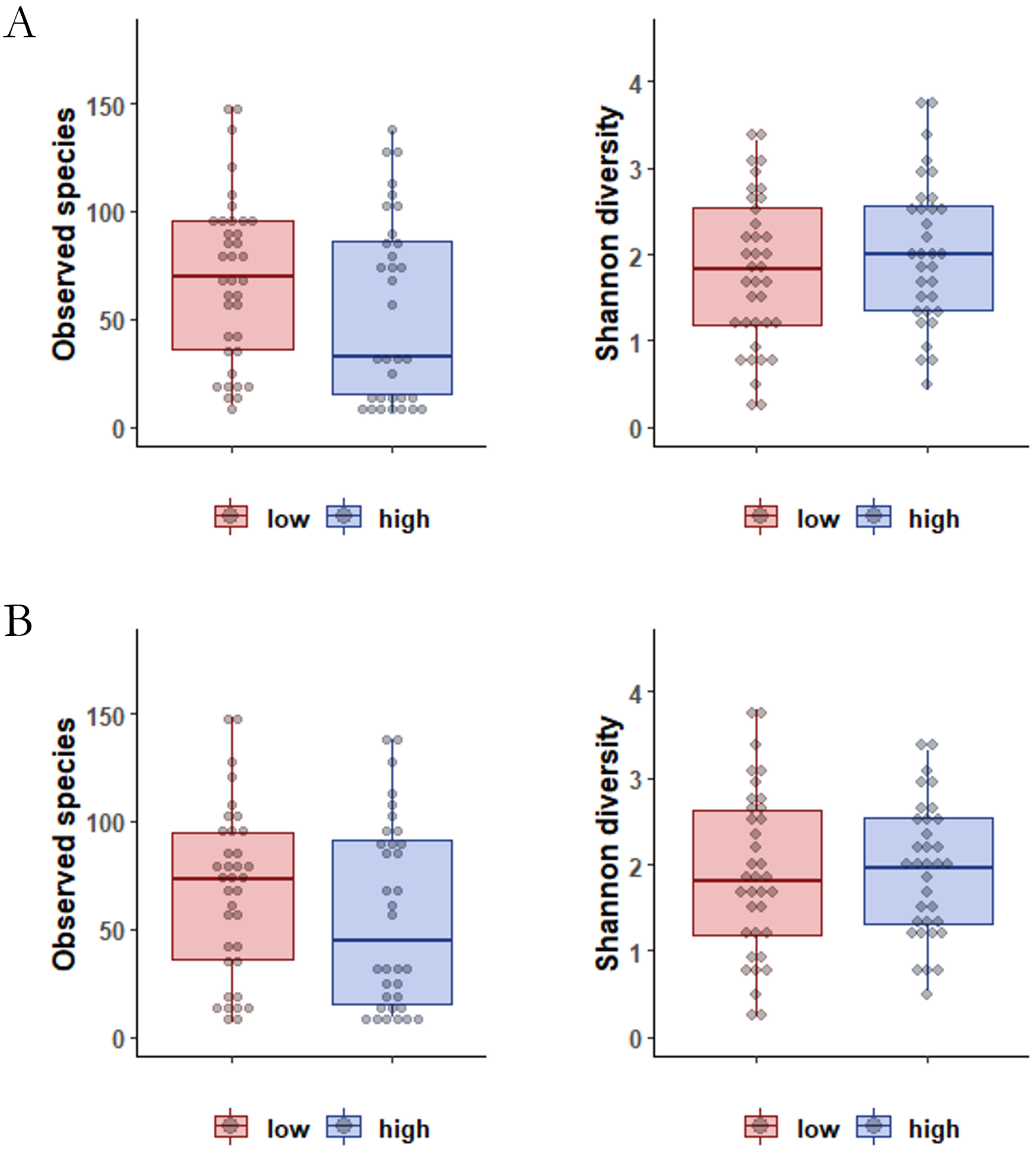
**

**eFigure 4. Assessment of lung microbiota alpha diversity in BAL fluid samples in the study cohort, as stratified by either low- or high-Crs, and low- or high-VR, respectively.** Measurements of alpha diversity observed species and Shannon diversity index allowed to assess differences in the lung microbiota alpha diversity between low-Crs and high-Crs patients (**Panel A**), as well as between low-VR and high-VR patients (**Panel B**), respectively. However, none significant difference (particularly for the Shannon diversity index) emerged at the Kruskal-Wallis test. [**Abbreviations.** Crs: compliance of the respiratory system; VR: ventilatory ratio; BAL: bronchoalveolar lavage].

**
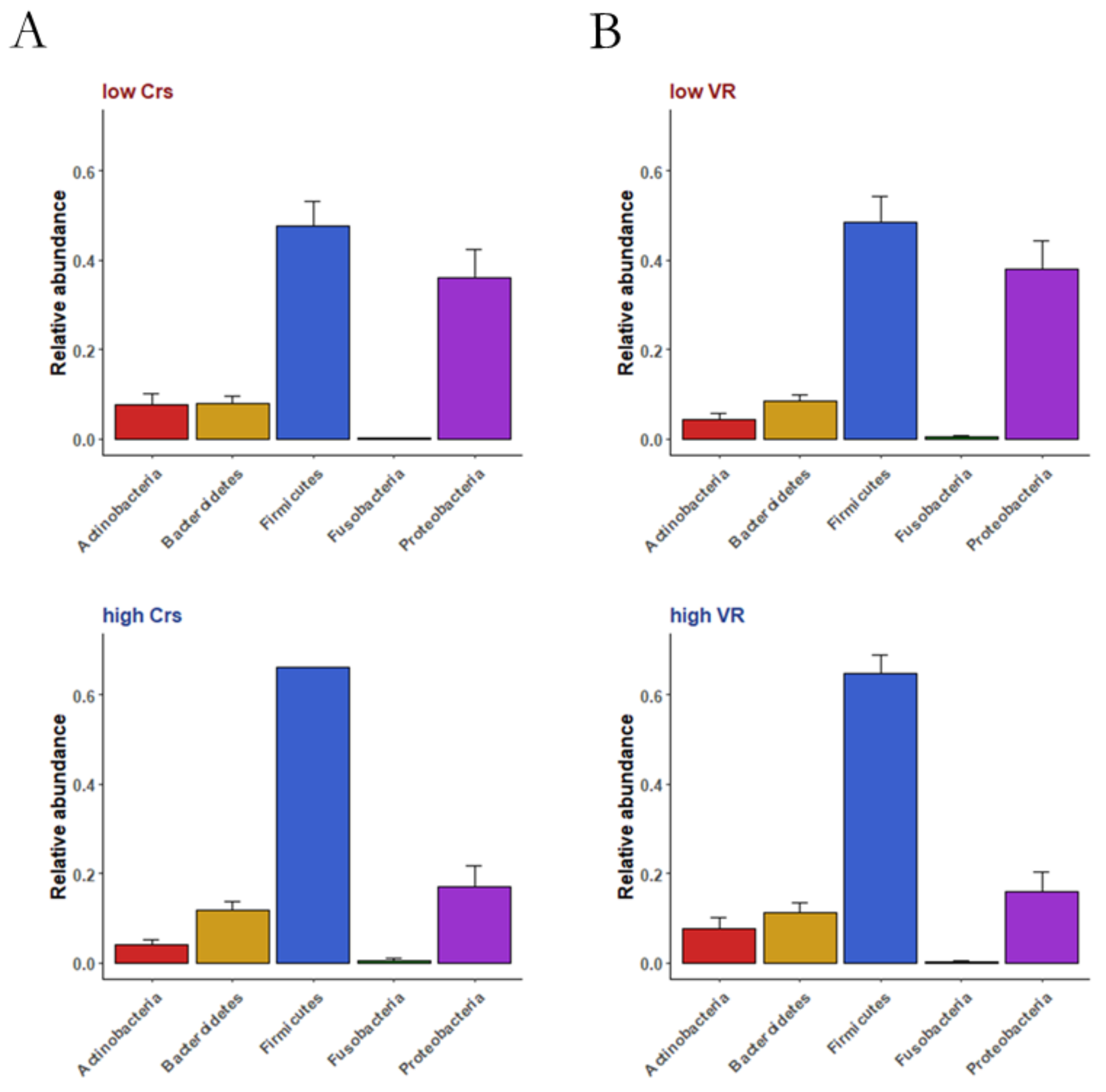
**

**eFigure 5. Assessment of lung microbiota composition at phylum level in BAL fluid samples from patients who had been categorized as low- or high-Crs, and low- or high-VR, respectively.** Comparison of low-Crs *vs* high-Crs patients’ samples showed statistically significant differences in the relative abundances of Firmicutes and Proteobacteria (Kruskal-Wallis test, p<0.05) (**Panel A**). Instead, the comparison of low-VR *vs* high-VR patients’ samples showed significant differences only for Proteobacteria (Kruskal-Wallis test, p<0.05) (**Panel B**). [**Abbreviations**. Crs: compliance of the respiratory system; VR: ventilatory ratio; BAL: bronchoalveolar lavage].

**
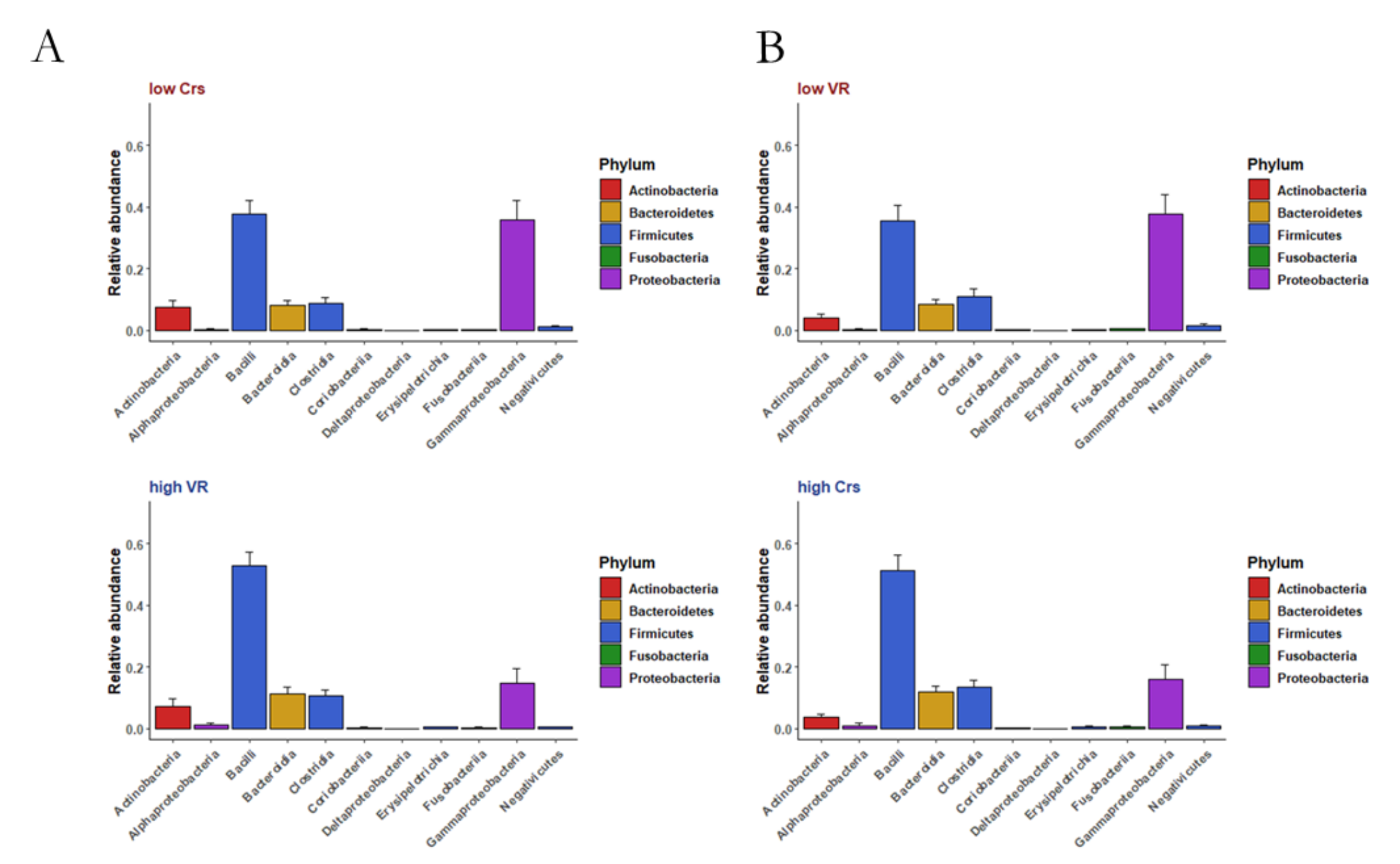
**

**eFigure 6: Assessment of lung microbiota composition at class level in BAL fluid samples from patients who had been categorized as low- or high-Crs, and low- or high-VR, respectively.** Comparison of low-Crs *vs* high-Crs patients’ samples showed statistically significant differences in the relative abundances of Bacilli and Gammaproteobacteria (Kruskal-Wallis test, p<0.05) (**Panel A**). Comparison of low-VR *vs* high-VR patients’ samples showed as well statistically significant differences in the relative abundances of Bacilli, Deltaproteobacteria, Fusobacteriia, and Gammaproteobacteria (Kruskal-Wallis test, p<0.05) (**Panel B**). [**Abbreviations**. Crs: compliance of the respiratory system; VR: ventilatory ratio; BAL: bronchoalveolar lavage].

**
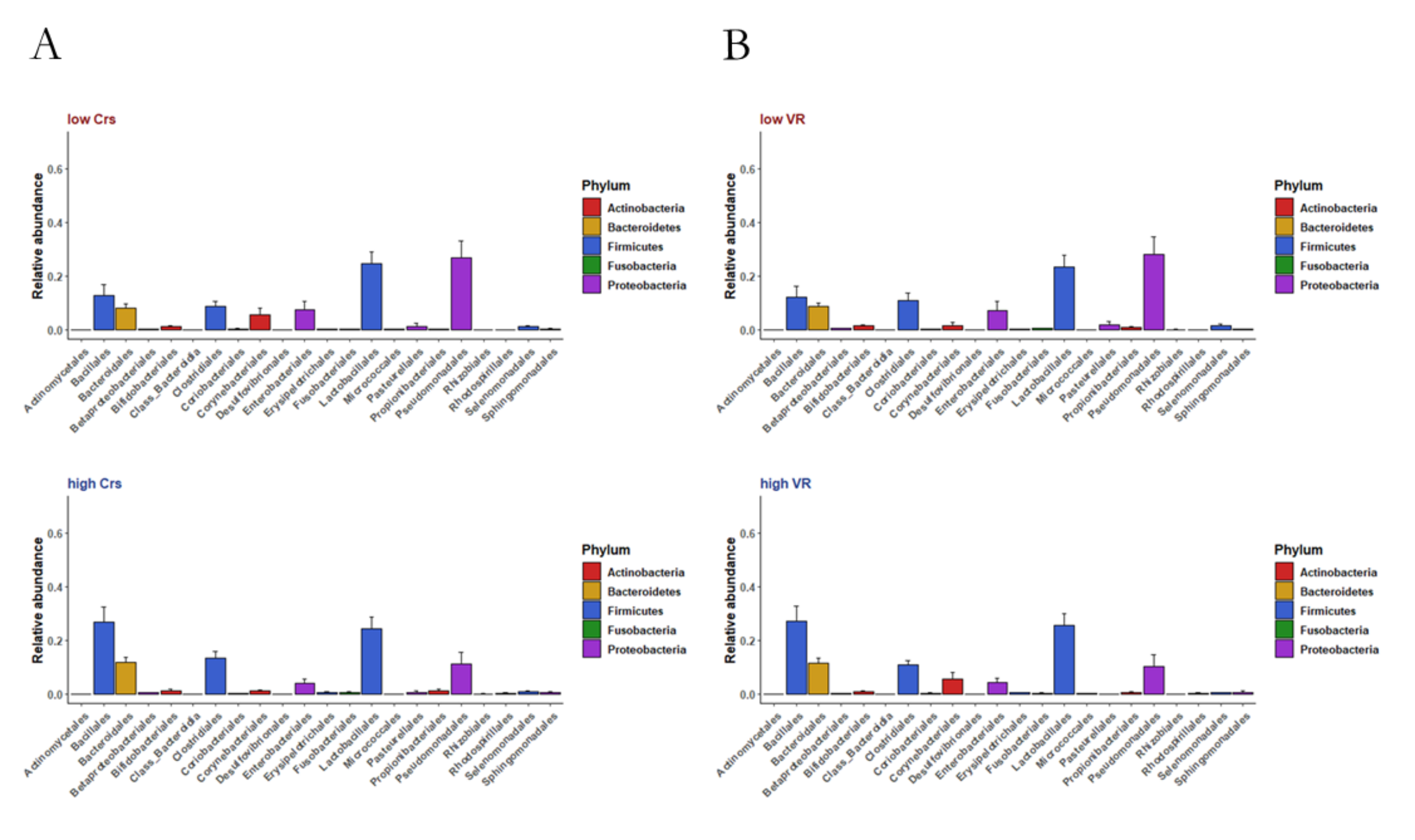
**

**eFigure 7. Assessment of lung microbiota composition at order level in BAL fluid samples from patients who had been categorized as low- or high-Crs, and low- or high-VR, respectively.** Comparison of low-Crs *vs* high-Crs patients’ samples showed statistically significant differences in the relative abundance of Bacillales (Kruskal-Wallis test, p<0.05) (**Panel A**). Comparison of low-VR *vs* high-VR patients’ samples showed as well statistically significant differences in the relative abundances of Desulfovibrionales and Fusobacteriales (Kruskal-Wallis test, p<0.05) (**Panel B**). [**Abbreviations**. Crs: compliance of the respiratory system; VR: ventilatory ratio; BAL: bronchoalveolar lavage].

**
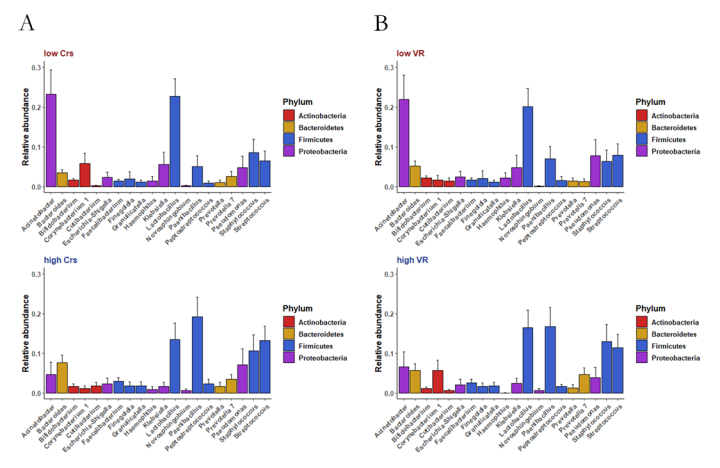
**

**eFigure 8. Assessment of lung microbiota composition at genus level in BAL fluid samples from patients who had been categorized as low- or high-Crs, and low- or high-VR, respectively.** Comparison of low-Crs *vs* high-Crs patients’ samples showed statistically significant differences in the relative abundances of Acinetobacter, Lactobacillus, and Paenibacillus (Kruskal-Wallis test, p<0.05) (**Panel A**). Comparison of low-VR *vs* high-VR patients’ samples showed instead statistically significant differences only in the relative abundance of Paenibacillus (Kruskal-Wallis test, p<0.05) (**Panel B**). [**Abbreviations**. Crs: compliance of the respiratory system; VR: ventilatory ratio; BAL: bronchoalveolar lavage].

**
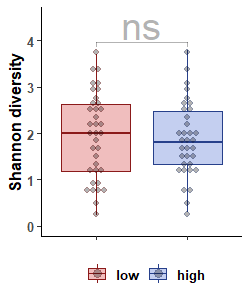

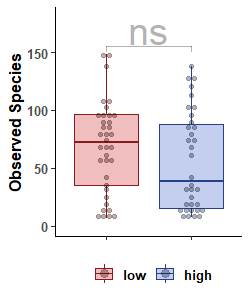
**
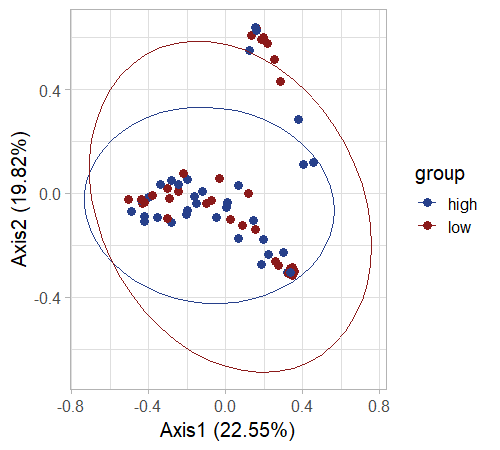


**
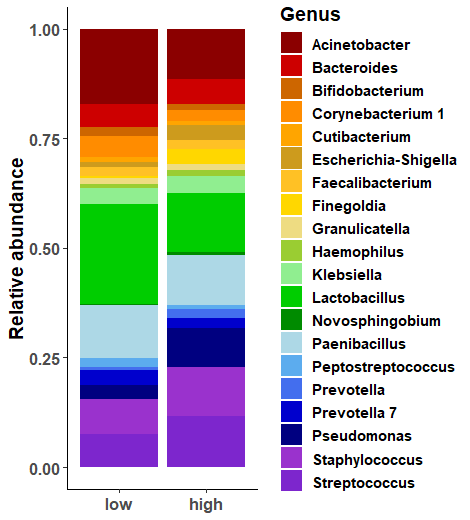
**
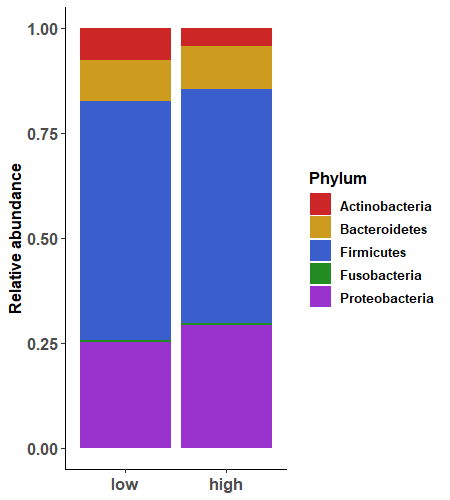


**eFigure 9: Comparison of community composition between SARS-CoV-2 infected patients admitted in ICU clustered according to the median value of OSI. Richness (observed species) and Shannon diversity were depicted and analysed by Wilcoxon test. Bray-Curtis beta diversity was measured and reported as Principal Coordinate analysis (PCoA). Permutational multivariate analysis of variance (PERMANOVA) was computed by using the adonis.2 function in vegan package with 1000 permutations. Relative abundances at Phylum and Genus (top 20) levels were reported.**

**
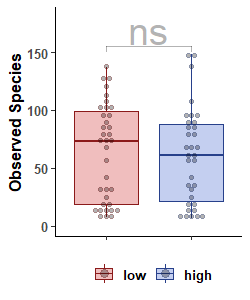
**
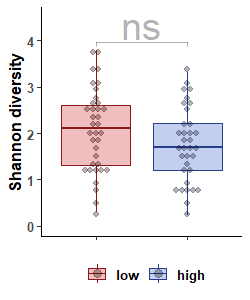


**
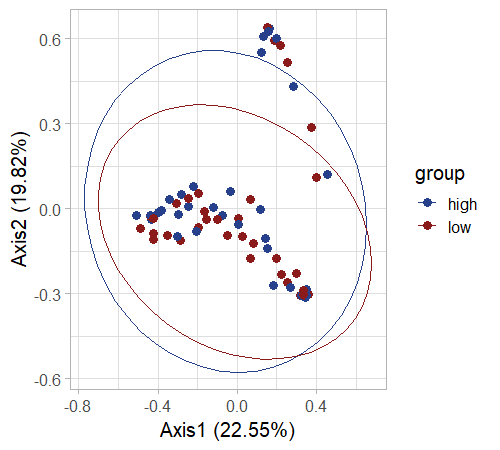
**

**
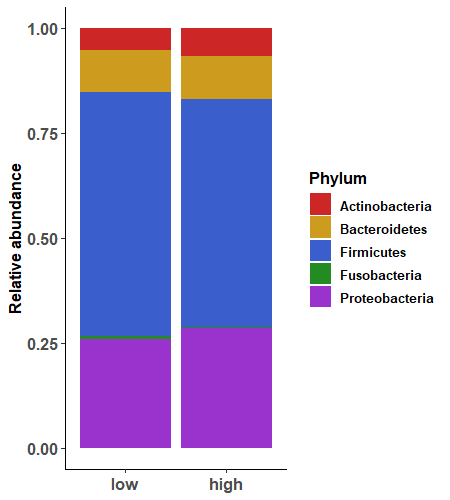
** **
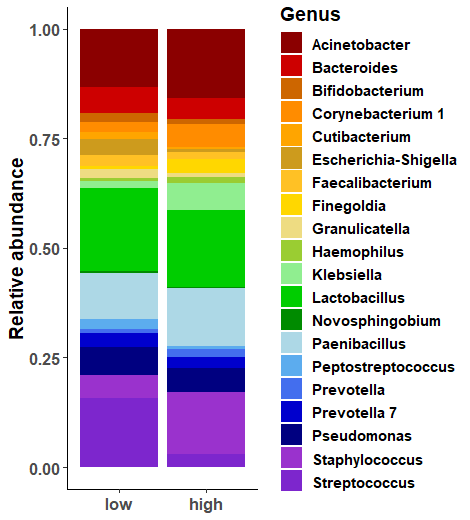
**

**eFigure 10: Comparison of community composition between SARS-CoV-2 infected patients admitted in ICU clustered according to the median value of DPRR. Richness (observed species) and Shannon diversity were depicted and analysed by Wilcoxon test. Bray-Curtis beta diversity was measured and reported as Principal Coordinate analysis (PCoA). Permutational multivariate analysis of variance (PERMANOVA) was computed by using the adonis.2 function in vegan package with 1000 permutations. Relative abundances at Phylum and Genus (top 20) levels were reported**

**References**

1. ARDS Definition Task Force, Ranieri VM, Rubenfeld GD, Thompson BT, et al. Acute respiratory distress syndrome: the Berlin Definition. JAMA. 2012; 307:2526-33. doi: 10.1001/jama.2012.5669.
2. Tang YW, Schmitz JE, Persing DH, Stratton CW. Laboratory diagnosis of COVID-19: current issues and challenges. J Clin Microbiol. 2020; 58:e00512-20. doi: 10.1128/JCM.00512-20.
3. Bello G, Bisanti A, Giammatteo V, et al. Microbiologic surveillance through subglottic secretion cultures during invasive mechanical ventilation: a prospective observational study. J Crit Care. 2020; 59:42-48. doi: 10.1016/j.jcrc.2020.05.013.
4. Kalil AC, Metersky ML, Klompas M, et al. Management of adults with hospital-acquired and ventilator-associated pneumonia: 2016 clinical practice guidelines by the Infectious Diseases Society of America and the American Thoracic Society. Clin Infect Dis. 2016; 63:e61-e111. doi: 10.1093/cid/ciw353.
5. Torres A, Niederman MS, Chastre J, et al. International ERS/ESICM/ESCMID/ALAT guidelines for the management of hospital-acquired pneumonia and ventilator-associated pneumonia: guidelines for the management of hospital-acquired pneumonia (HAP)/ventilator-associated pneumonia (VAP) of the European Respiratory Society (ERS), European Society of Intensive Care Medicine (ESICM), European Society of Clinical Microbiology and Infectious Diseases (ESCMID) and Asociación Latinoamericana del Tórax (ALAT). Eur Respir J. 2017; 50:1700582. doi: 10.1183/13993003.00582-2017.
6. Gertler R. Respiratory mechanics. Anesthesiol Clin. 2021; 39:415–440. doi: 10.1016/j.anclin.2021.04.003.
7. Kallet RH, Zhuo H, Liu KD, Calfee CS, Matthay MA; National Heart Lung and Blood Institute ARDS Network Investigators. The association between physiologic dead-space fraction and mortality in subjects with ARDS enrolled in a prospective multi-center clinical trial. Respir Care. 2014; 59:1611–1618. doi: 10.4187/respcare.02593.
8. Monteiro ACC, Vangala S, Wick KD, et al. The prognostic value of early measures of the ventilatory ratio in the ARDS ROSE trial. Crit Care. 2022; 26:297. doi: 10.1186/s13054-022-04179-7.
9. De Pascale G, De Maio F, Carelli S, et al. *Staphylococcus aureus* ventilator-associated pneumonia in patients with COVID-19: clinical features and potential inference with lung dysbiosis. Crit Care. 2021; 25:197. doi: 10.1186/s13054-021-03623-4.
10. Pathak JL, Yan Y, Zhang Q, Wang L, Ge L. The role of oral microbiome in respiratory health and diseases. Respir Med. 2021; 185:106475. doi: 10.1016/j.rmed.2021.106475.
11. Dickson RP. The microbiome and critical illness. Lancet Respir Med. 2016; 4:59–72. doi: 10.1016/S2213-2600(15)00427-0.
12. Bassis CM, Erb-Downward JR, Dickson RP, et al. Analysis of the upper respiratory tract microbiotas as the source of the lung and gastric microbiotas in healthy individuals. mBio. 2015; 6:e00037. doi: 10.1128/mBio.00037-15.
13. Eisenhofer R, Minich JJ, Marotz C, Cooper A, Knight R, Weyrich LS. Contamination in low microbial biomass microbiome studies: issues and recommendations. Trends Microbiol. 2019; 27:105–17. doi: 10.1016/j.tim.2018.11.003
14. Stecher B, Chaffron S, Käppeli R, et al. Like will to like: abundances of closely related species can predict susceptibility to intestinal colonization by pathogenic and commensal bacteria. PLoS Pathog. 2010; 6:e1000711. doi: 10.1371/journal.ppat.1000711.
15. De Maio F, Posteraro B, Ponziani FR, Cattani P, Gasbarrini A, Sanguinetti M. Nasopharyngeal microbiota profiling of SARS-CoV-2 infected patients. Biol Proced Online. 2020; 22:18. doi: 10.1186/s12575-020-00131-7.
16. Bolyen E, Rideout JR, Dillon MR, et al. Reproducible, interactive, scalable and extensible microbiome data science using QIIME 2. Nat Biotechnol. 2019; 37:852–7. doi: 10.1038/s41587-019-0209-9.
17. Callahan BJ, McMurdie PJ, Rosen MJ, Han AW, Johnson AJ, Holmes SP. DADA2: high-resolution sample inference from Illumina amplicon data. Nat Methods. 2016; 13:581–3. doi: 10.1038/nmeth.3869.
18. Bokulich NA, Kaehler BD, Rideout JR, et al. Optimizing taxonomic classification of marker-gene amplicon sequences with QIIME 2's q2-feature-classifier plugin. Microbiome. 2018; 6:90. doi: 10.1186/s40168-018-0470-z.
19. McMurdie PJ, Holmes S. phyloseq: an R package for reproducible interactive analysis and graphics of microbiome census data. PLoS One. 2013; 8:e61217. doi: 10.1371/journal.pone.0061217.
20. Davis NM, Proctor DM, Holmes SP, Relman DA, Callahan BJ. Simple statistical identification and removal of contaminant sequences in marker-gene and metagenomics data. Microbiome. 2018; 6:226. doi: 10.1186/s40168-018-0605-2.
21. Oksanen J, Simpson G, Blanchet F, Kindt R, Legendre P, Minchin P, O'Hara R, Solymos P, Stevens M, Szoecs E, Wagner H, Barbour M, Bedward M, Bolker B, Borcard D, Carvalho G, Chirico M, De Caceres M, Durand S, Evangelista H, FitzJohn R, Friendly M, Furneaux B, Hannigan G, Hill M, Lahti L, McGlinn D, Ouellette M, Ribeiro Cunha E, Smith T, Stier A, Ter Braak C, Weedon J (2022). _vegan: Community Ecology Package_. R package version 2.6-4, <https://CRAN.R-project.org/package=vegan>.
22. R Core Team. R: a language and environment for statistical computing. R Foundation for Statistical Computing; Vienna, Austria: 2022. [(last accessed on June 30, 2023)]. Available at: https://www.R-project.org/.
23. Wickham H. ggplot2: elegant graphics for data analysis. Springer; New York, NY, USA: 2016.
24. Kassambara A, Kosinski M, Biecek P (2021). _survminer: Drawing Survival Curves using 'ggplot2'_. R package version 0.4.9, <https://CRAN.R-project.org/package=survminer>. [(last accessed on June 30, 2023)].
25. Therneau T (2023). _A Package for Survival Analysis in R_. R package version 3.5-5, <https://CRAN.R-project.org/package=survival>. [(last accessed on June 30, 2023)]. Available at: http://CRAN.R-project.org/package=survival.
26. Schemper M. Cox analysis of survival data with non-proportional hazard functions. J R Stat Soc Ser D. 1992; 41:455–65. doi: 10.2307/2349009.
27. Schemper M., Wakounig S., Heinze G. The estimation of average hazard ratios by weighted Cox regression. Stat Med 2009; 28:2473–89. doi: 10.1002/sim.3623.
28. Therneau T., Grambsch P. Modeling survival data: extending the Cox model. Springer; New York, NY, USA: 2000. [Google Scholar].
29. Dunkler D., Ploner M., Schemper M., Heinze G. Weighted Cox regression using the R package coxphw. J Stat Softw 2018; 84:1–26. doi: 10.18637/jss.v084.i02.
30. Hothorn T (2017). maxstat: maximally selected rank statistics. R package version 0.7-25. Available at: https://CRAN.R-project.org/package=maxstat.
31. Peduzzi P, Concato J, Feinstein AR, Holford TR. Importance of events per independent variable in proportional hazards regression analysis. II. Accuracy and precision of regression estimates. J Clin Epidemiol. 1995; 48:1503–10. doi:10.1016/0895-4356(95)00048-8.
